# Conifer: clonal tree inference for tumor heterogeneity with single-cell and bulk sequencing data

**DOI:** 10.1186/s12859-021-04338-7

**Published:** 2021-08-30

**Authors:** Leila Baghaarabani, Sama Goliaei, Mohammad-Hadi Foroughmand-Araabi, Seyed Peyman Shariatpanahi, Bahram Goliaei

**Affiliations:** 1 Institute of Biochemistry and Biophysics, University of Tehran, Tehran, Iran; 2 Faculty of New Sciences and Technologies, University of Tehran, Tehran, Iran; 3 Department of Mathematical Sciences, Sharif University of Technology, Tehran, Iran

**Keywords:** Heterogeneity of tumor, Clonal tree, Bulk sequencing, Single-cell sequencing, Bayesian nonparametric model

## Abstract

**Background:**

Genetic heterogeneity of a cancer tumor that develops during clonal evolution is one of the reasons for cancer treatment failure, by increasing the chance of drug resistance. Clones are cell populations with different genotypes, resulting from differences in somatic mutations that occur and accumulate during cancer development. An appropriate approach for identifying clones is determining the variant allele frequency of mutations that occurred in the tumor. Although bulk sequencing data can be used to provide that information, the frequencies are not informative enough for identifying different clones with the same prevalence and their evolutionary relationships. On the other hand, single-cell sequencing data provides valuable information about branching events in the evolution of a cancerous tumor. However, the temporal order of mutations may be determined with ambiguities using only single-cell data, while variant allele frequencies from bulk sequencing data can provide beneficial information for inferring the temporal order of mutations with fewer ambiguities.

**Result:**

In this study, a new method called Conifer (ClONal tree Inference For hEterogeneity of tumoR) is proposed which combines aggregated variant allele frequency from bulk sequencing data with branching event information from single-cell sequencing data to more accurately identify clones and their evolutionary relationships. It is proven that the accuracy of clone identification and clonal tree inference is increased by using Conifer compared to other existing methods on various sets of simulated data. In addition, it is discussed that the evolutionary tree provided by Conifer on real cancer data sets is highly consistent with information in both bulk and single-cell data.

**Conclusions:**

In this study, we have provided an accurate and robust method to identify clones of tumor heterogeneity and their evolutionary history by combining single-cell and bulk sequencing data.

**Supplementary Information:**

The online version contains supplementary material available at 10.1186/s12859-021-04338-7.

## Background

Genetic mutation is a major cause of abnormal cell growth and cancer. Although cancer cells are usually derived from one mutated cell initially and therefore share mutated genes, new mutations may happen in cancer development [1]. In other words, cancer cells in a tumor are not homogeneous and tumor genomic heterogeneity is shown in many studies [1–3]. A tumor consists of different clones, each of which is a set of cells sharing a common genotype inherited from a common ancestor [4]. In order to understand individual tumors heterogeneity and their phylogenetic inference which can probably be a helpful component for personalized cancer treatment, it is critical to properly identify their clones, determine the development stage of cancer cells and identify early single-nucleotide variants (SNVs) that have led to rapid cell growth [5–7].

Sequencing of bulk data that focuses on DNA of a mixture of thousands or millions of cancerous and/or normal cells is widely used for providing a mixed signal of variant allele frequencies (VAFs) for each somatic mutation. In order to discover the evolutionary history, bulk sequencing data needs deconvolution analysis [8], which often includes two successive deduction steps. At the first step, co-occurred SNV clusters are deduced by deconvolving the mixed signal of the bulk sample [9]. Afterward, the evolutionary relationship between clusters is deduced by using SNV cluster frequencies [10]. However, in some methods such as PhyloWGS [11], these two inference steps are carried out jointly to avoid SNV clusters that are phylogenetically incompatible. In most tumor heterogeneity analyses based on bulk sequencing data such as PyClone [9], PhyloSub [12], Clomial [13], and AncesTree [14], it is generally supposed that SNVs with similar VAFs belong to the same clone.

It is shown in various cases that only relying on frequencies observed in a bulk sample may not be enough to infer the evolutionary history, and taking multiple samples is required [8]. In addition, the assumption that SNVs with similar frequencies belong to the same clone may be violated, since a tumor may be composed of clones with similar frequencies but different genotypes.

Moreover, even though low-frequency SNVs are common and can play a decisive role in tumor diversity, the process of their prevalence discovery from bulk sequencing is not accurate enough [15].

To achieve higher resolution for inferring evolutionary history, single-cell sequencing was introduced, which allows direct acquisition of cell genotypes without the need to deconvolution of mixed signals [16–20], and resulted in reducing the possibility of ignoring low-frequency SNVs. In addition, single-cell information about co-occurred SNVs can be used to differentiate between clusters of SNVs with the same prevalence [21].

Single-cell sequencing is well used in methods like SCITE [22], OncoNEM [23], and SiFit [24] to infer mutational trees, though clonal frequencies are not reported in them. Furthermore, in SiCloneFit [25], a nonparametric Bayesian mixture model based on a Chinese Restaurant Process (CRP) is introduced to infer the clonal genotypes and their evolution based on a finite-sites model. Although SiCloneFit has an assumption in clonal tree inference that each node of the tree can only have up to two children (binary tree), however the polytomies of the binary clonal tree can be inferred by removing the branches which are unsupported by mutations in a post-processing step. On the other hand, Conifer can directly infer a tumor phylogeny with polytomy by the joint modeling of bulk and single-cell sequencing data.

Despite all of its advantages, the single-cell sequencing approach is costly and error-prone. False-positive errors occur due to the DNA amplification error, and false-negative errors occur due to the missing of one or both alleles (dropout). Furthermore, another type of noise may occur in data as a result of accidental sequencing of two or more cells.

Considering the advantages and disadvantages of bulk and single-cell sequencing data, the idea of utilizing both data types is used in several studies, to reduce inaccuracies in each approach and consequently achieve more accurate clonal tree inference. As an example of the benefits of this combination, the temporal order of mutations can be determined using decreasing VAFs from bulk sequencing data with fewer ambiguities than using only single-cell data. On the other hand, branching events in the evolutionary tree can be inferred more accurately with single-cell data.

ddClone [26] analyzes intra-tumor heterogeneity using single-cell and bulk sequencing data and proposes a probabilistic model based on the nonparametric Bayesian method to deduce tumor clones. The prior of the Bayesian method is obtained from single-cell data, and the likelihood is obtained from bulk sequencing data. However, ddClone does not infer tumor phylogeny and is not sufficient for understanding cancer tumor evolution.

B-SCITE [21] is the first computational approach that infers tumor phylogeny from combined single-cell and bulk sequencing data. This probabilistic method searches for tumor phylogenetic trees to maximize the joint likelihood of the two data types. In this method, tree search is carried out with a customized Markov chain Monte Carlo (MCMC) algorithm over the space of labeled trees [21]. B-SCITE is mainly designed for inferring mutational trees and straightforward clonal tree inference is not provided.

In addition, PhISCS [27] is a combinatorial approach that uses integer linear programming for mutational tree inference based on single-cell and bulk sequencing data. However, PhISCS does not infer tumor subclones and their evolutionary relationship directly.

In this study, we have proposed a new method Conifer, which utilizes both single-cell and bulk sequencing data to infer tumor clones and their evolutionary relationship. In contrast to SiCloneFit, there is no limitation on the depth and number of branches of the inferred tree of Conifer. In Conifer single-cell sequencing data is used to resolve the challenge of identifying similar prevalent clones in the tumor and to resolve ambiguities in the phylogeny inference. On the other hand, Conifer uses bulk sequencing data to reduce the negative effects of sampling biases and false-negative mutations. Based on our knowledge, Conifer is the first method that introduces the tumor clonal tree using both single-cell and bulk sequencing data.

As clones and their evolutionary tree are not predefined, Conifer provides a Bayesian nonparametric model and a tree-structure Chinese Restaurant Process (CRP) is used as its prior. To approximate the posterior of the Bayesian model, the particular MCMC algorithm that Conifer performs is a Collapsed Gibbs Sampling in which some of the latent variables are marginalized out to speed up the coverage of the chain. As a result, Conifer introduces a clonal tree in which each node represents the clonal genotypes that have occurred together and are shared between different cells. It is noteworthy that Conifer employs the infinite sites assumption (ISA) which implies that mutations persist once occurred. In nodes closer to the tree root, corresponding clonal genotypes are shared between a larger number of cells, while moving away from the root and towards the leaves, clones become more specialized to particular cells in those paths.

We evaluated Conifer performance in clone identification comprehensively on various simulated datasets with different numbers of clones, bulk and single-cell samples, etc. and compared it with the best methods such as B-SCITE [21] and ddClone [26] in the field, based on V-measure [28] and adjusted rand score [29] criteria. Moreover, Conifer introduces the clonal evolutionary trees on simulated data which are compared with methods like B-SCITE [21], OncoNEM [23], and PhyloWGS [11] based on co-clustering accuracy and ancestor–descendant accuracy criteria (the definition of these criteria are given in [21]). In conclusion, Conifer has higher accuracy in clone identification and phylogeny inference than other existing methods in most cases. In addition, Conifer is more evaluated on real data sets of cancer by comparing its evolutionary tree with other methods. By deep investigation, it is shown that Conifer evolutionary tree is completely consistent with VAFs of mutations in bulk data and also co-occurrence of mutations in single-cell data.

## Results and discussion

**Fig. 1:**
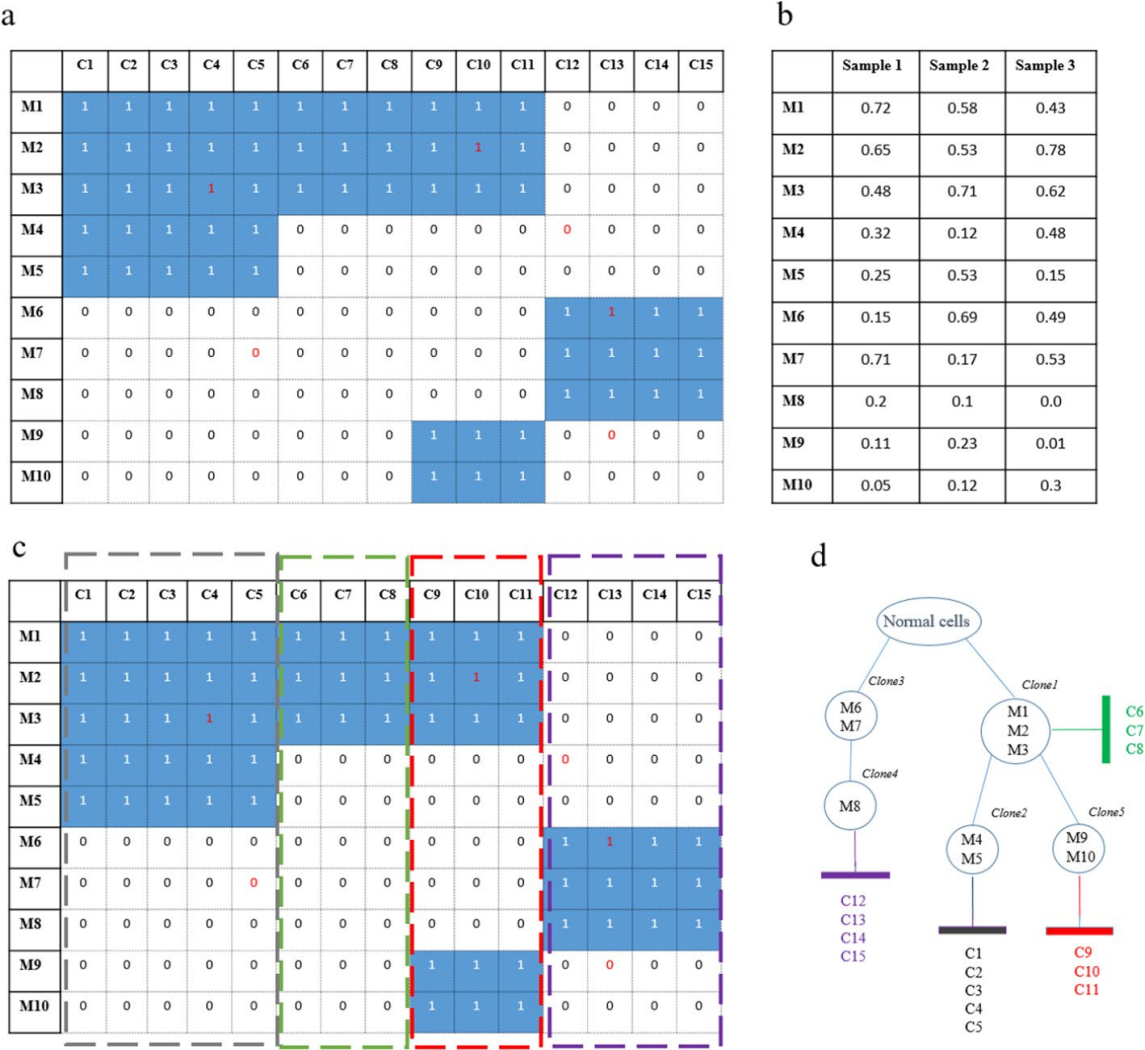
Schematic representation of combining single-cell and bulk sequencing data for clonal tree inference in Conifer method, **a** $n \times m$ matrix in which each row and column represents SNVs and cell, respectively. White elements show no mutation and blue ones show mutation has occurred. 1 and 0 with the red font show false-positive and false-negative (drop-out events), respectively, **b** $n \times b$ matrix that its rows are SNVs and its columns are bulk samples and $B_{ij}$ is variant allele frequency in bulk samples, **c** co-occurred patterns of SNVs in single-cell profiles which are determined by dashed rectangles, **d** the inferred clonal tree and cell attachment

Conifer uses mutation co-occurrence information in single-cell data for inferring tree branching alongside the VAFs in bulk sequencing data for identifying clones and their temporal ordering. In Fig. 1, it is shown schematically how two data types are connected to infer the clonal tree. As it is shown in Fig. 1a, single-cell data is represented as a matrix with rows and columns showing SNVs and cells, respectively, and each element indicating the presence or absence of corresponding SNV in a cell. Moreover, bulk sequencing data is considered as a matrix in which each element presents VAFs related to SNV in different bulk sequencing samples (Fig. 1b).

In the Conifer method, it is assumed that SNVs with similar VAFs in different bulk samples most likely belong to a common cluster, unless there is no single-cell showing two SNVs have co-occurred. All co-occurred patterns in the single-cell profile are considered as the prior knowledge for mutation clustering. In Fig. 1c, the co-occurred patterns are shown as dashed rectangles. Using these patterns, the clonal tree with attached single-cell samples can be inferred, with the mutations of each pattern presented in each of its path (Fig. 1d). Clustering technique and phylogenetic inference are described in detail in the “Material and method” section.

### Performance on simulated data

Since the clonal tree is not known for data of real cancer tumors, a complete set of data is simulated and used to evaluate the performance of Conifer. To simulate data, the idea of ddClone [26] and B-SCITE [21] studies are used (see Additional file 1 for details). The simulated data covers various cell counts (25,50,100 and 500), numbers of clones (10, 15, 20, and 40), and different types of error in single-cell data. The root node of each clonal tree represents a healthy cell population, and SNVs are randomly distributed between other nodes.

For evaluating the accuracy of the Conifer inferred clonal tree, it is compared with B-SCITE [21], OncoNEM [23], and PhyloWGS [11] according to the co-clustering accuracy measure. B-SCITE uses both single-cell and bulk sequencing data while OncoNEM and PhyloWGS use only single-cell and only bulk sequencing data, respectively.

In addition, the ancestor–descendant accuracy measure is used for comparing the Conifer tree with B-SCITE in the presence of copy number variation (CNV). The plots are generated by ggplot2 [30] to illustrate the accuracy of the methods on different criteria.

Additionally, for evaluating the accuracy of clustering, the V-measure [28] and the adjusted rand score [29] criteria are used which are implemented in scikit-learn Python package 0.19.2. Their corresponding scores are between 0 and 1, which 0 represents random labeling independent of the number of clusters and 1 shows the accurate clustering.

In order to measure the method sensitivity respecting errors in single-cell sequencing data, different types of errors such as assortment bias and doublet rate are examined and explained in Additional file 1. Assortment bias error is indicated by the parameter $\uplambda$, large values of which present less assortment bias and equivalently less difference between single-cell and bulk genotype prevalence.

#### Clonal tree accuracy

**Fig. 2:**
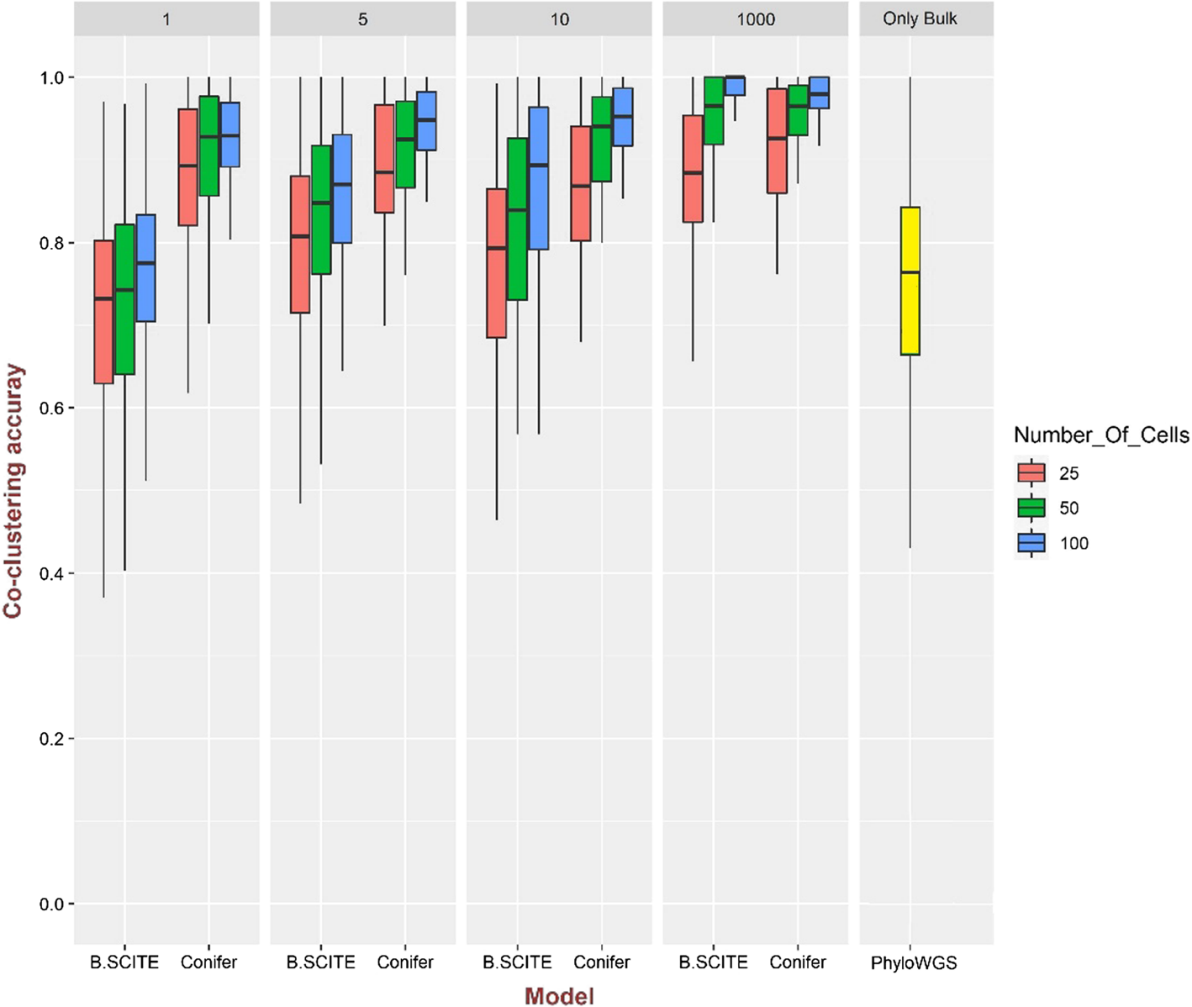
Comparison of co-clustering accuracy in B-SCITE, Conifer, and PhyloWGS models for 100 clonal trees simulated with 10 clones and 50 mutations and for $\lambda$ = 1, 5, 10 and 1000. For single-cell data 25, 50, and 100 genotypes are extracted for each clonal tree. There are two bulk sequencing samples with a coverage of 10,000. The following errors are added to the single-cell data: the false-positive rate of 10^–5^, the false-negative rate of 0.2, the missing rate of 0.05, and the doublet rate of 0.1

**Fig. 3:**
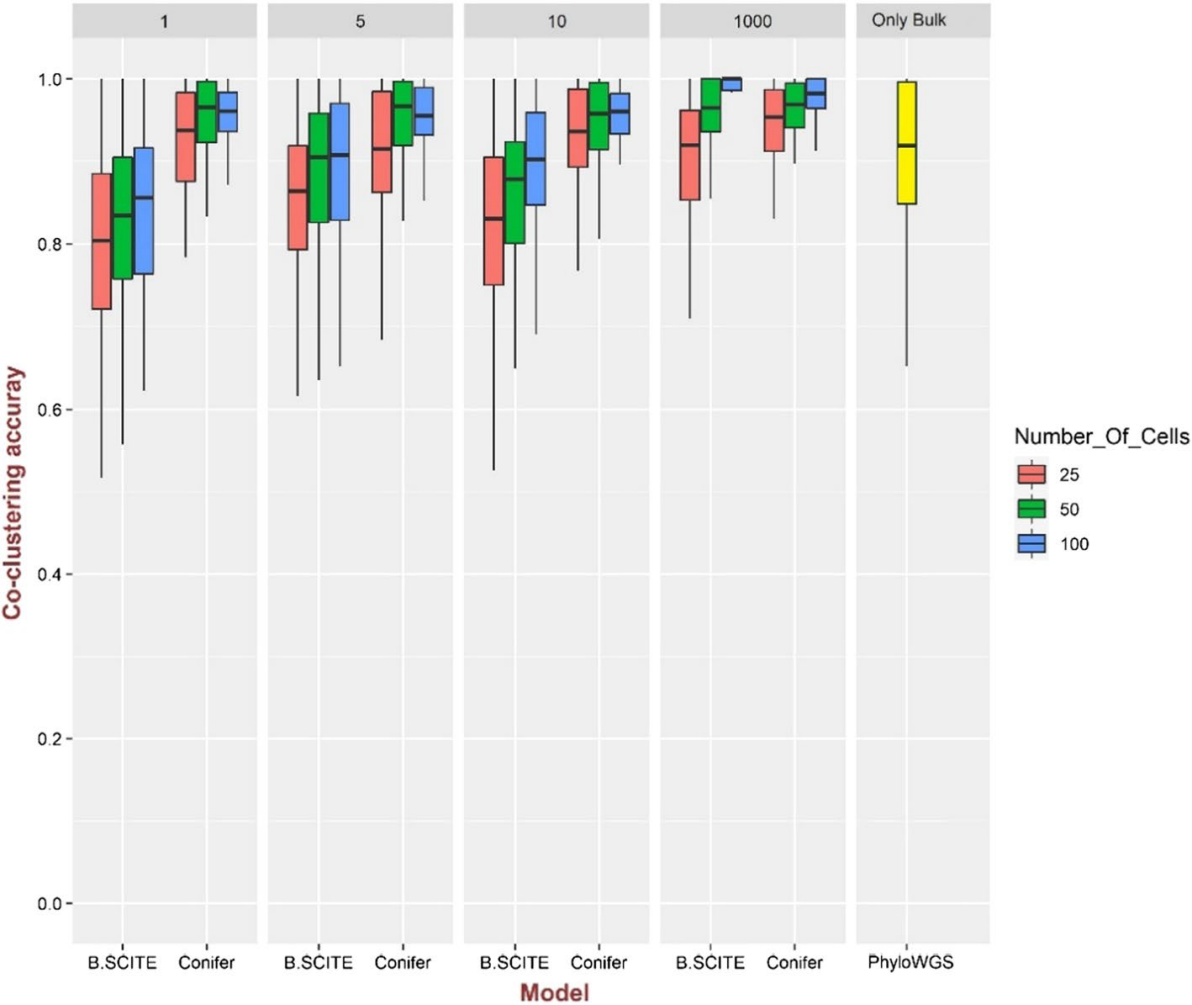
Comparison of co-clustering accuracy in B-SCITE, Conifer, and PhyloWGS models for 100 clonal trees simulated with 10 clones and 50 mutations. For $\lambda$ = 1, 5, 10 and 1000. For single-cell data 25, 50, and 100 genotypes are extracted for each clonal tree. The number of bulk sequencing samples is 2 with a coverage of 10,000. The following errors are added to the single-cell data: the false-positive rate of 10^–5^, the false-negative rate of 0.2, the missing rate of 0.05, and the doublet rate of 0.1

Comparison of Conifer with B-SCITE and PhyloWGS methods using the co-clustering accuracy measure is shown in Figs. 2 and 3 for 100 simulated clonal trees with 10 clones and 25, 50, and 100 numbers of cells and 1 and 2 bulk sequencing samples with coverage of 10,000. Simulated single-cell data which is used by both Conifer and B-SCITE is generated with the false-positive rate of ${10}^{-5}$, the false-negative rate of 0.2, the missing rate of 0.05, and the doublet rate of 0.1 with various ranges of values for $\uplambda$ ($\uplambda \hspace{0.17em}$= 1, 5, 10, and 1000), while PhyloWGS method uses only bulk sequencing data. Instead of only mutations which are common between single-cell and bulk sequencing data, all mutations in bulk data are considered in this comparison. As it is shown in Fig. 2 for one bulk sample and Fig. 3 for two bulk samples, although the performance of all three methods is improved by increasing the number of bulk samples, Conifer shows the highest accuracy for different single-cell sequencing data and different numbers of bulk samples. In addition, in both Conifer and B-SCITE methods, for a specific number of bulk samples, the accuracy is increased by increasing the number of single cells. Also, the accuracy of B-SCITE is decreased by decreasing the value of $\uplambda$ while Conifer is almost stable for different values of $\uplambda$. Additionally, it should be noted that although the doublet rate is not considered in the Conifer model, it is accurate with doublet rate of 0.1 according to the results shown in Fig. 2.

**Fig. 4:**
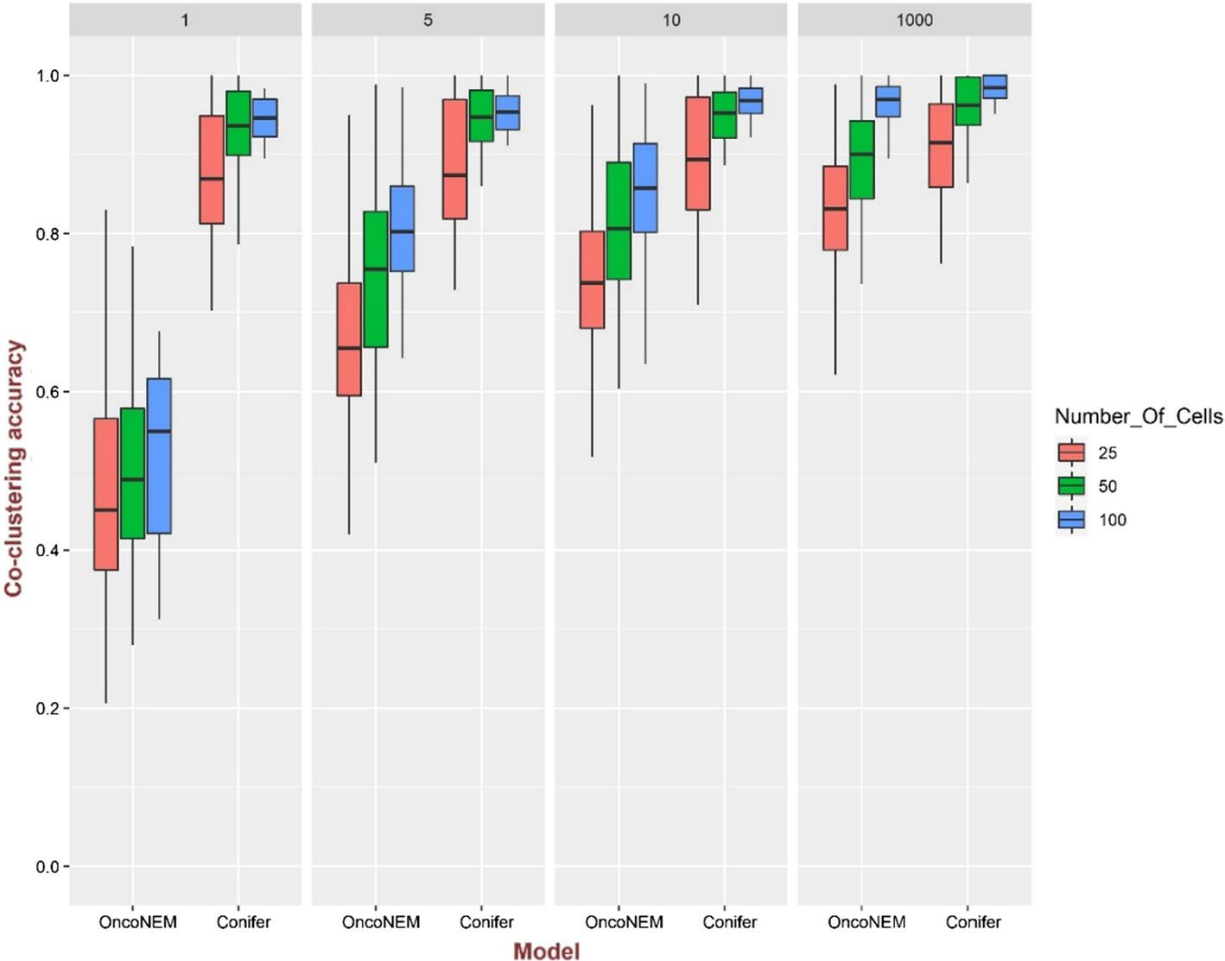
Comparison of co-clustering accuracy in OncoNEM and Conifer models for 100 clonal trees simulated with 20 clones and 100 mutations and for $\lambda$ = 1, 5, 10 and 1000. For single-cell data 25, 50, and 100 genotypes are extracted for each clonal tree. There is one bulk sequencing sample with a coverage of 10,000. The following errors are added to the single-cell data: the false-positive rate of 10^–5^, the false-negative rate of 0.2, the missing rate of 0.05, and the doublet rate of 0.1

The above comparison is repeated between Conifer and OncoNEM and shown in Fig. 4. As OncoNEM uses only single-cell data, only SNVs which are common between single-cell and bulk sequencing data are considered in Conifer to make the comparison meaningful. Conifer is run with one bulk sequencing sample and shows better accuracy than OncoNEM for various ranges of values for $\uplambda$. Moreover, improved performance of OncoNEM for larger values of $\uplambda$ together with the high accuracy of Conifer even for small values of $\uplambda$ is evidence for the effectiveness of Conifer’s approach in combining single-cell and bulk sequencing data, as large values of λ indicate less difference between single-cell and bulk genotype frequencies.

#### The presence of CNV

Although Conifer assumes that SNVs are obtained from the copy-number-neutral regions and the VAFs are not affected by copy number variations, it is still accurate regarding the alterations of CNV, on account of the fact that single-cell sequencing data is also used for clone identification and tree inference. VAF alterations by CNV can result in the incorrect inference of mutation clustering in models that only rely on VAFs similarities in bulk sequencing data.

Comparisons of the ancestor–descendant accuracy measure for Conifer and B-SCITE on two simulated data sets with 30% and 50% of CNV are shown in Additional file 1: Figs. S1 and S2, respectively. It can be concluded that the accuracy of Conifer is not affected significantly by changing the proportion of CNVs and it is more accurate than B-SCITE for various numbers of cells in both data sets.

#### Clone identification accuracy

Accuracy of Conifer, ddClone, and B-SCITE methods in SNV clustering is evaluated and compared in Additional file 1: Figs. S3 and S4, for 100 clonal trees simulated with 10, 20, and 40 clones, 100 SNVs, one bulk sequencing sample with coverage of 10,000, and 50 single-cell genotypes. Simulated single-cell data is generated with the following errors: the false-positive rate of ${10}^{-5}$, the false-negative rate of 0.2, the missing rate of 0.05, and the doublet rate of 0.1. In this comparison, the mutations that are not present in single-cell sequencing data are ignored. In Additional file 1: Fig. S3, Conifer has outperformed both methods in various numbers of clones and various ranges of values for λ according to adjusted rand score measure. In Additional file 1: Fig. S4, both Conifer and B-SCITE perform very well in detecting the correct clones according to the V-measure, while ddClone presents lower accuracy. As it can be concluded from Additional file 1: Fig. S4, the results of all three methods are not highly sensitive to different values of λ.

#### Large false-positive rates

The performance of Conifer is more evaluated for larger values of false-positive rates. The inferred trees of Conifer, B-SCITE, and OncoNEM are compared in Additional file 1: Fig. S5 for the false-positive rate of 1%. With this relatively large false-positive rate the co-clustering accuracy is slightly decreased for all three methods, however, Conifer's accuracy is still in an acceptable range. By increasing the value of λ the accuracy of all methods improves and Conifer has the largest accuracy among them for λ = 1000. In addition, the clustering accuracy is compared between Conifer and ddClone using the V-measure accuracy for larger values of false-positive rate of 5% and 10%. In Additional file 1: Fig. S6 it is shown that although the V-measure accuracy is moderately decreased by larger values of false-positive rates, Conifer is still accurate enough and outperforms ddClone. However, this accuracy decrease is because false-positive errors can violate the assumed co-occurrence of mutations which is used by Conifer as prior knowledge in clustering of SNVs based on their VAFs.

### Performance on real data

#### Colorectal cancer

Conifer performance is further evaluated on real data of a patient (CRC2) with colorectal cancer which is provided in the study of Leung et al. [31]. It is noteworthy that in this dataset there are two bulk sequencing data of primary and metastatic tumors together with single-cell sequencing data.

For CRC2 patient, 182 cells are sequenced from the primary colorectal and liver metastatic tumors. The number of SNVs reported by the original study is 36. Genotypes reported as binary values indicate the presence or absence of mutation in an SNV locus. In this study, cells with no mutation are ignored and 25 SNVs and 86 cells are considered for CRC2 patient.

**Fig. 5:**
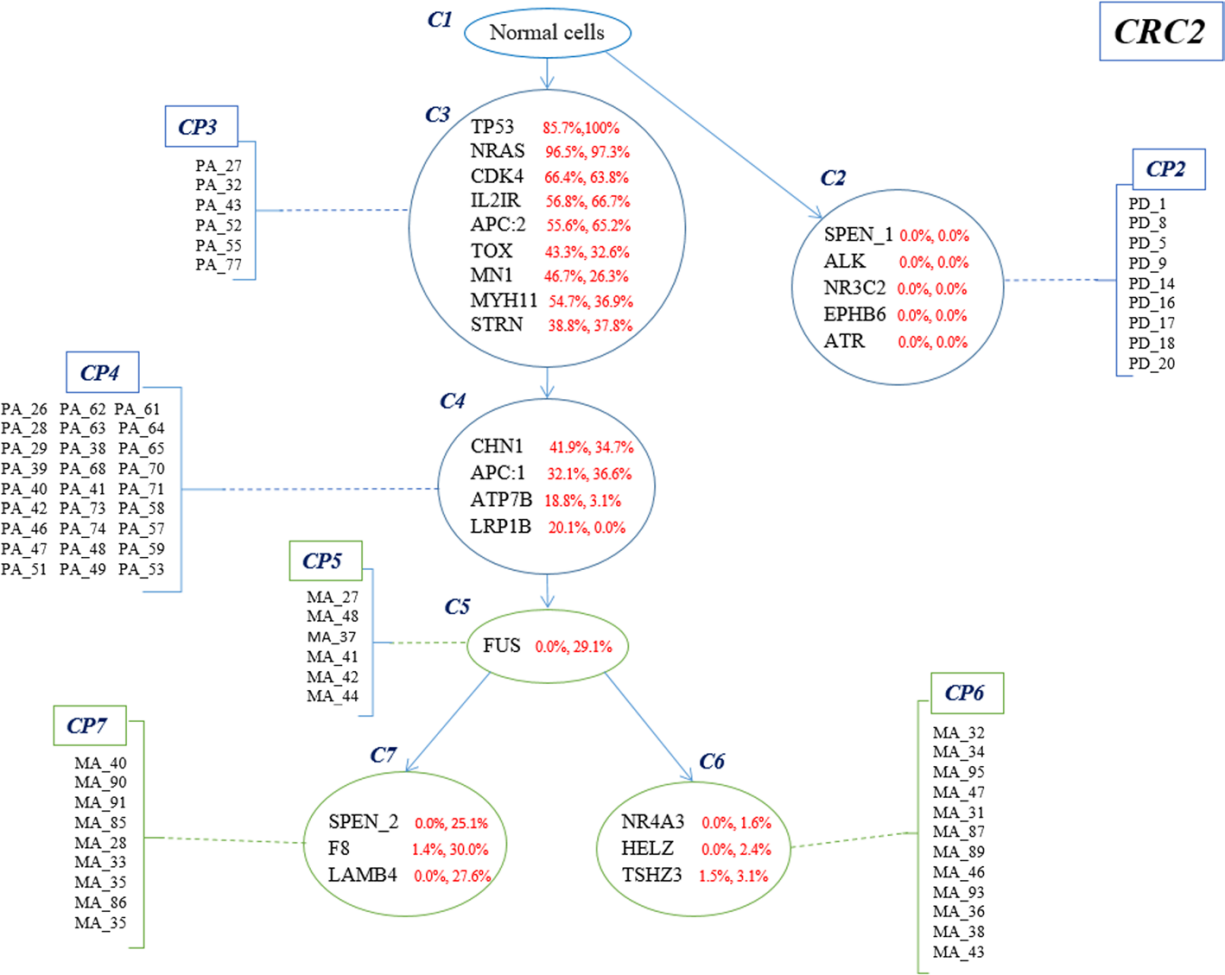
Clonal evolution tree inferred by Conifer for CRC2 patient tumor data. For each SNV, two numbers are reported: VAFs in colorectal tumor bulk sample and metastasis liver bulk sample

The clonal tree inferred by Conifer for this dataset is shown in Fig. 5. Each branch in the tree represents the mutation profile of one or a set of cells, and each clone is a set of mutations that have occurred in a branch and their VAF frequencies are similar in different bulk sequencing samples.

Conifer method introduces a tree with 7 nodes (clones) so that the root node is for the non-mutant genotype (C1) and two nodes C2 and C3 are its descendants. C2 is a cluster that contains somatic mutations (SPEN_1, NR3C2, EPHB6, ATR). It is different from primary and metastatic tumor clones and has a separate branch in the clonal tree. This clone and its separated branch are also mentioned in the original study of Leung et al. [31]. C3 is the first evolved clone from healthy cells and has nine mutations, including tp53 which is a tumor suppressor gene. The cell population CP3 is attached to C3 clonal genotype.

In the evolutionary process after formation of the C3 clone and before tumor metastasis, the C4 clone is formed with mutations (CHN1, ATP7B, APC: 1, LRP1B) and CP4 cell population. This clone is introduced as a result of VAFs similarity and mutation occurrence in one single-cell profile. In the original study of Leung et al. [31], SCITE method [22] is used to infer the evolutionary tree of mutation, and two distinct branches for metastatic cells are reported based on single-cell sequencing data, assuming that the mutations are not missed during evolution.

Based on both bulk and single-cell sequencing data, Conifer method shows that a group of cells of the primary clone C4 has migrated to the liver, and this migration has occurred only once. Conifer concludes that the migrated cells are subjected to the FUS mutation in the liver, creating the clone C5 and then evolved into two separate branches. The reason that Conifer represents the FUS mutation as a separate clone is although the FUS mutation should belong to the clone C6 considering neighboring mutations with close frequencies based on single-cell data, as the VAF is not similar to other mutations in that clone (NR4A3, HELZ, TSHZ3), a separate cluster is created. As it is shown in Fig. 5, Conifer concludes that in addition to the clone C6, the clone C5 is also the ancestor of the clone C7, which can be explained by the false positives that occurred in the profiles of eight cells. In fact, it indicates the possibility of co-occurrence of FUS mutation with mutations of clone C7 (SPEN_2, F8, LAMB4).

Comparison of the Conifer inferred tree and the clonal tree introduced in SiCloneFit [25], which is based on only single-cell sequencing data, shows some worth-mentioning differences. In SiCloneFit [25] two “IL2IR” and “APC: 2” mutations are co-occurred in the first clone of the primary tumor. On the contrary, Conifer concludes that they belong to the second clone of the primary tumor according to the similarity of those two mutations VAFs. Additionally, the clonal tree of SiCloneFit [25] for patient CRC2 represents polyclonal seeding. In other words, it shows the existence of two distinct branches for metastasis. In fact, in SiCloneFit it is concluded that two distinct groups of cells with different mutations have migrated from the primary clone and formed two independent metastatic clones, and the FUS mutation has occurred in both of them independently and during two different evolutionary processes. However, Conifer inferred tree is more likely to be consistent with VAFs similarity as the VAF value of FUS mutation in the metastatic sample (29.1) is approximately equal to the total mean VAF value of C6 (2.36) and C7 (27.56). A recent study that proposes a method named SCARLET [32] also shows monoclonal seeding by investigating changes in copy number variation of single-cell sequencing data, which corroborates the Conifer’s tree.

#### Triple-negative breast cancer (TNBC)

**Fig. 6:**
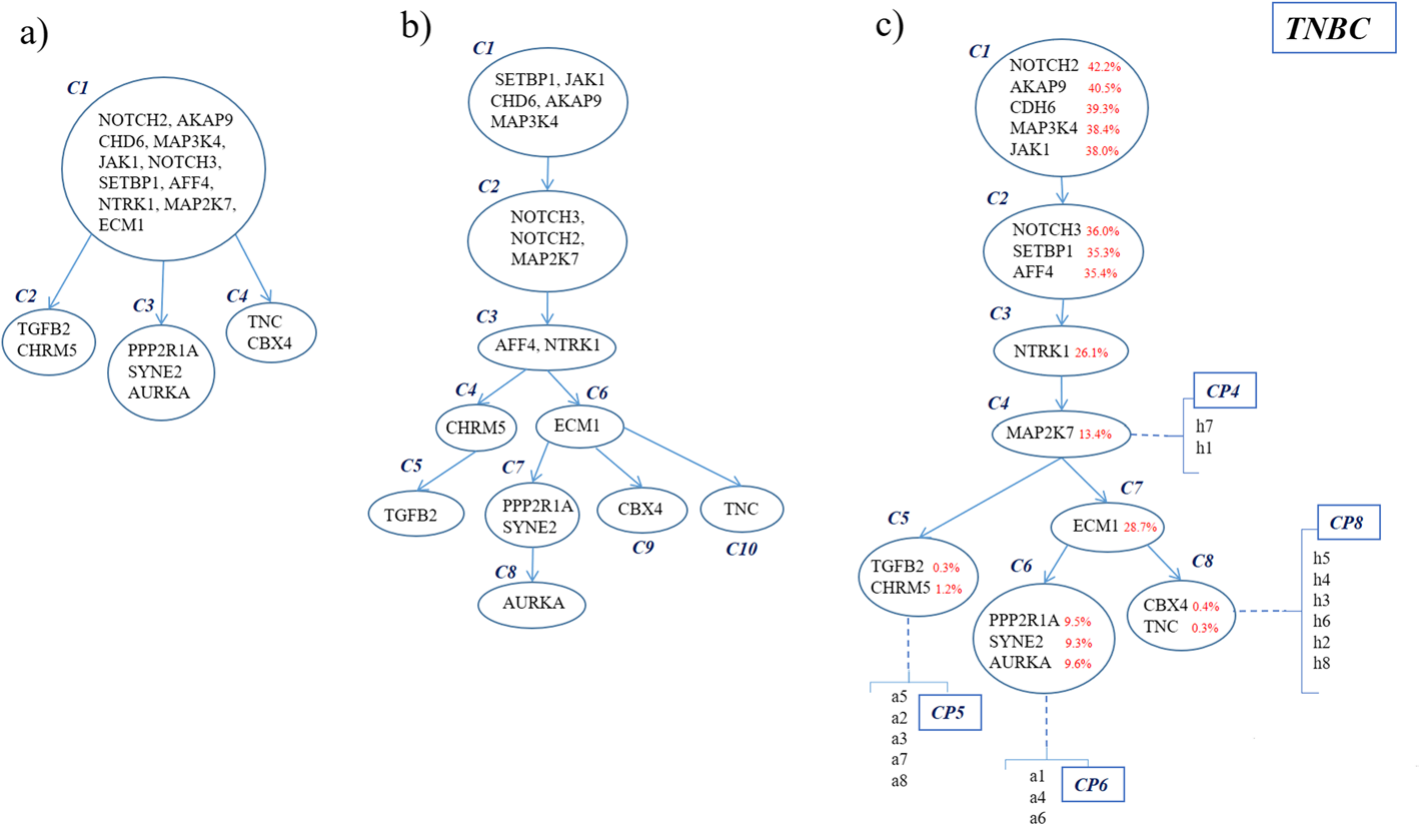
Clonal tree inference for a patient with triple-negative breast cancer, **a** Clonal tree Inferred in the original study [33] based on single-cell exome and copy number data, **b** Clonal tree Inferred by PhISCS based on single-cell data, **c** Clonal tree inferred by Conifer based on bulk and single-cell data

The performance of Conifer is more evaluated on real data by analyzing the triple-negative breast cancer in the study of Wang et al. [33]. Single-cell profiles of 16 cells were provided after performing copy number profiling and exome sequencing. Clonal trees inferred by the original study, PhISCS [27], and Conifer for selected 18 mutations (with coverage of ${10}^{5}$), are shown in Fig. 6. Conifer provides clonal tree based on bulk and single-cell data while clonal evolution in the original study is inferred based on single-cell exome and copy number data, and PhISCS has taken the matrix of single-cell sequencing data and the estimated noise rates of SCS experiment as its inputs (the clonal tree is reported in the study of Karpov et al. [34]).

Mutations which are presented in the linear part of all trees (before branching) are quite similar to each other except for the mutation ECM1 which is discussed in the following.

As it is shown in Fig. 6c, Conifer introduces 4 different clones C1, C2, C3, and C4 for those mutations which all belong to clone C1 in the original study. This difference is because the mutations in the clone C1 of the original study have different VAFs and as Conifer uses bulk sequencing VAFs together with single-cell data, it proposes different clustering which is more consistent with mutations VAFs similarity.

In addition, clones of the mutations SETBP1 with the VAF value of 35.3% and NOTCH2 with the VAF value of 42.2% are different between Conifer and PhISCS. In PhISCS, they belong to clones C1 and C2, respectively while in Conifer they are clustered oppositely. In other words, in the Conifer tree, the NOTCH2 mutation belongs to the clone C1 with the mean VAF value of 39.05% and the SETBP1 mutation belongs to the clone C2 with the mean VAF value of 35.7%. Moreover, in contrast to PhISCS in which the mutation AFF4 with the VAF value of 35.4% belongs to the clone C3 (which also contains the mutation NTRK1 with the VAF value of 26.1%), Conifer has put it in the same clone of the mutations SETBP1 and NOTCH3 with the VAF values of 35.3% and 36%, respectively which is more consistent with similarity of VAFs. The difference in placement of the MAP2K7 mutation between Conifer and PhISCS is also worth mentioning. Conifer puts the mutation MAP2K7 with the VAF value of 13.4% in a distinct clone C4 while in PhISCS tree it is in the same clone of the mutations NOTCH2 and NOTCH3 with VAF values of 42.2% and 36% which are significantly different with VAF value of the mutation MAP2K7 and seems to be inconsistent with VAF similarity.

Single-cell co-occurrence frequencies of the mutation ECM1 are not similar to mutations of clone C5 (TGFB2, CHRM5), therefore ECM1 and C5 (TGFB2, CHRM5) are placed in different branches in Conifer. On the other hand, the mutation ECM1 is similar to mutations in clones C6 and C8 regarding single-cell co-occurrence frequencies and consequently, Conifer introduces the clone C7 (containing the mutation ECM1) as the ancestor of clones C6 and C8. The placement of this mutation clearly shows how Conifer relies on VAFs similarity data for identifying clones and simultaneously uses single-cell data for finding the most appropriate place for clones in the tree.

Finally, mutations in leaf clones of the original study tree (clones C2, C3, and C4) are quite similar to mutations in leaf clones of the Conifer tree (clones C5, C6, and C8) and slightly different from the PhISCS tree in which if clone pairs (C4, C5), (C7, C8) and (C9, C10) are formed according to VAF similarity and then clones of each pair are merged, the resulted clones will be equivalent to clones C2, C3 and C4 in the original study tree, respectively.

## Conclusion

In this study, a new reliable and effective method named Conifer is introduced for inferring tumor clonal tree by combining single-cell and bulk sequencing data. Conifer provides a generative nonparametric model for the identification of clones and their evolutionary relationship based on single-cell and bulk sequencing data by considering infinite site assumption (ISA).

Conifer method has the distinctive feature of simultaneously identifying both clones and phylogenetic tree. Each tree branch contains mutations of one or more cells, and their common clones are obtained by their VAFs similarity in different bulk sequencing samples. In the Conifer inferred tree, clones with genotypes that are common in more cells are closer to the root.

In order to evaluate the performance of Conifer, comprehensive sets of single-cell and bulk sequencing data are simulated with varying numbers of SNVs, cells, bulk samples, and clones. Additionally, a wide range of error rates, assortment biases, and doublets are considered. By studying the simulated datasets, it is shown that Conifer is more accurate than other existing methods on different criteria for evaluation of clone identification and clonal evolutionary tree. For assessing Conifer performance on real datasets, data of a patient with colorectal cancer is used. In this investigation, Conifer provides the genotype of clones, cell population, and clonal tree by considering combined single-cell and bulk sequencing data of the primary and metastatic tumors. In the obtained clonal tree, the evolutionary stage in which the metastasis has occurred is clearly identified.

Additionally, the performance of Conifer is assessed on real data of a patient with triple-negative breast cancer and it is shown that Conifer inferred tree is completely consistent with VAFs and co-occurrence of mutations in bulk and single-cell data, respectively.

In conclusion, Conifer provides a more accurate clonal tree of tumor heterogeneity comparing to other existing methods. It is achieved by combining single-cell and bulk sequencing data, the former of which is used for resolving the challenge of identifying similar prevalent clones that co-occur in the tumor and, resolving the ambiguities of phylogeny inference, while the latter is used to reduce the effects of single-cell sequencing errors such as false-negative rates and sampling biases.

## Material and method

Conifer aims to introduce a rooted clonal tree $T$ with $s$ nodes which are labeled as $N\left( T \right) = \left\{ {\vartheta_{0} ,\vartheta_{1} , \ldots ,\vartheta_{s} } \right\}$, by using single-cell mutation profiles and VAFs of different bulk sequencing samples. Conifer provides a Bayesian nonparametric model for inferring clonal trees without any knowledge of clones or their evolutionary tree. In the Bayesian nonparametric model, the posterior distribution on infinite collections of tree hierarchies and clones must be found. The inference of posterior distribution is performed by the MCMC algorithm for approximating distributions over trees, clones, and SNVs allocations.

Two main steps of the algorithm are sampling path assignments and sampling level allocations, which are repeated successively for a sufficient number of iterations so that the Markov chain converges to the stationary distribution. These two steps are explained in this section.

The nested CRP introduced in the study of Blei et al. [35] is used as a prior of the Bayesian nonparametric model provided by Conifer. In addition, the nested CRP model is extended by Conifer in such a way that instead of ordinary CRP, distance-dependent CRP [36] is used for level allocations. In order to review the main components of Conifer, they are briefly explained below.

### Nested CRP

It is a process for providing a prior on tree topologies without any limitation on its width and depth. To understand nested CRP, the Chinese Restaurant Process (CRP) should be defined first. The CRP is a stochastic process for introducing the distribution of customers, which sequentially enter a restaurant with infinite tables and sit at a table. The probability of sitting at a table is proportional to the number of customers who have already sat at that table. Customers can also sit at a new table with probability proportional to the model parameter $\gamma$. The formed sitting plans represent customer clustering. For showing the formulation of CRP, the notation in the study of Blei et al. [36] is used. The table assignment for customer $i$ is $c_{i}$, and at the time of customer $i$’s entrance, $K$ tables are occupied by customers $1$ to $i - 1$. Assume $n_{k}$ is the number of customers sitting at table $k$, then the $c_{i}$ is drawn as Eq. (1).

1 $$p\left( {c_{i} = k{|}c_{{1:\left( {i - 1} \right)}} ,\gamma } \right) \propto \left\{ {\begin{array}{*{20}l} {n_{k} } \hfill & {for \quad k \le K} \hfill \\ \gamma \hfill & {for \quad k = K + 1} \hfill \\ \end{array} } \right.$$

The nested CRP is an extended CRP in which instead of having only one restaurant, it is assumed that there is an infinite number of Chinese restaurants with an infinite number of tables. A restaurant is selected as the root, on each table of which, there is a card with the name of the next restaurant, to which those sitting on that table should go the next night. In fact, as each restaurant is referred to only once, so the relations between different restaurants form a tree structure. Therefore, the nested CRP provides a prior on tree topologies and each node of the tree provides a CRP over its descendant.

### Distance-dependent CRP

This process is a different representation of CRP in such a way that instead of joining different tables, customers join each other. Distance-dependent CRP [36] implies that if two customers have access to each other through a series of customer connections, then they are sitting at the same table. Therefore, the customers' seating assignment depends on the distances between them. For representing customer connections, a graph is defined in which nodes and edges represent customers and their connections, respectively. In other words, if $z_{i}$ is the index of a customer joining the customer $i$, then the binary $\left( {i,z_{i} } \right)$ is the directional graph edge. The clusters are defined according to connected sub-graphs in this similarity graph. Let ${\varvec{t}}$ and $f$ be the distance measurements between customers and the decay function, respectively. The customer assignments are drawn by distance-dependent CRP as Eq. (2).

2 $$p\left( {z_{i} = j{|}t,\eta } \right) \propto \left\{ {\begin{array}{*{20}l} {f\left( {t_{ij} } \right)} \hfill & { if\quad i \ne j } \hfill \\ \eta \hfill & { if\quad i = j} \hfill \\ \end{array} } \right.$$

The range of $i$ and $j$ is from 1 to the number of customers, and $\eta$ is the model parameter that controls the self-loop in the connectivity graph. Additionally, the induced table assignment is denoted by $l\left( z \right)$.

### Input data

Single-cell data is presented by a $n \times m$ matrix $M$ in which each row and column represents SNV and cell, respectively, and its elements with a value of zero show that no mutation has occurred in the corresponding position, while the value of one means a mutation has occurred.

In addition, bulk data is presented by a $n \times b{ }$ matrix $B$ that its rows are SNVs and its columns are bulk samples, and each element $B_{ij}$ is a variant allele frequency that corresponds to the ith SNV in the jth bulk sample.

### Sampling path

At the first iteration, Conifer uses nested CRP for generating a tree path in such a way that each cell is considered as a customer. To represent each single-cell $i$, ${\varvec{w}}_{d}$ is defined as a set of SNVs with the value of one in that cell. The process of generating path with nested CRP is as follows: at the first step (the first night in nested CRP) the root node $\vartheta_{0}$ does not have any child, therefore for ${\varvec{w}}_{1}$ (first customer) it generates node $\vartheta_{1}$ with the probability of one. This process repeats for $k$ steps (nights) and generates $k$ levels which $k$ is a random number limited to the size of ${\varvec{w}}_{1}$. The path corresponding to ${\varvec{w}}_{1}$ is labeled by ${\varvec{c}}_{1}$. For generating the path of the next cells (${\varvec{w}}_{d}$ with $d > 1$), there are two options; generating a node as a new child of the root node $\vartheta_{0}$ with probability of $\frac{\gamma }{{\gamma + d - 1{ }}}$ ($\gamma$ is the model parameter), or choosing child $\vartheta_{j}$ of the root node $\vartheta_{0}$ with probability of $\frac{{\left| {n_{j} } \right|}}{{\gamma + d - 1{ }}}$ ($\left| {n_{j} } \right|$ is the number of cells that have chosen $\vartheta_{j}$ so far) to go through. After processing each ${\varvec{w}}_{d}$ and generating its corresponding path ${\varvec{c}}_{d}$, its mutations are assigned to nodes of the path ${\varvec{c}}_{d}$ randomly. The probability of the generated tree is calculated by multiplying the probabilities of all paths and is used as the prior probability of the Bayesian model.

For the sampling path step of the next iterations, Conifer removes the corresponding path of each ${\varvec{w}}_{d}$ with its mutations from the tree and deletes possible empty nodes. Afterward, the removed path is added to the tree with the same procedure explained above (except the random assignment of mutations).

In addition, for those SNVs which are not present in any single cell, Conifer adds extra sets with one mutation and follows the same procedure for generating their path.

**Fig. 7:**
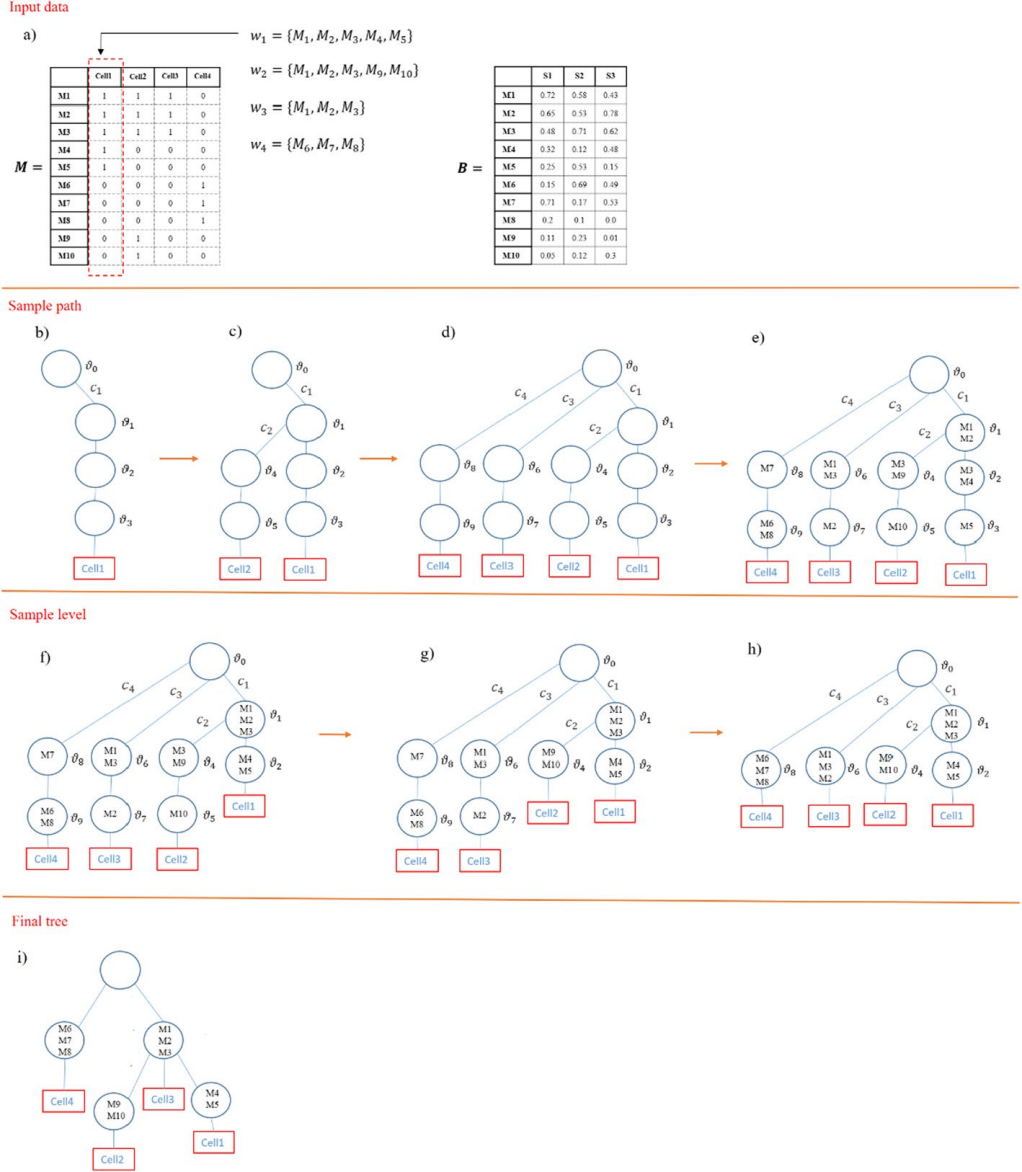
A schematic example showing sampling steps of tree inference by Conifer, **a** the variables ${\varvec{w}}_{1}$,${ }{\varvec{w}}_{2}$,${ }{\varvec{w}}_{3}$ and ${\varvec{w}}_{4}$ are defined for cells 1 to 4 which are sets of SNVs with the value of one in corresponding cells. Matrices ${\varvec{A}}$ and ${\varvec{B}}$ show the single-cell data and VAFs of SNVs in different bulk samples, respectively, **b** the generated path ${\varvec{c}}_{1}$ corresponding to ${\varvec{w}}_{1}$ with node labels of $\left\{ {\vartheta_{0} ,\vartheta_{1} ,\vartheta_{2} ,\vartheta_{3} } \right\}$, **c** the generated path ${\varvec{c}}_{2}$ corresponding to ${\varvec{w}}_{2}$ with node labels of $\left\{ {\vartheta_{0} ,\vartheta_{1} ,\vartheta_{4} ,\vartheta_{5} } \right\}$, **d** the generated paths ${\varvec{c}}_{3}$ and ${\varvec{c}}_{4}$ corresponding to ${\varvec{w}}_{3}$ and ${\varvec{w}}_{4}$ with node labels of $\left\{ {\vartheta_{0} ,\vartheta_{6} ,\vartheta_{7} } \right\}$ and $\left\{ {\vartheta_{0} ,\vartheta_{8} ,\vartheta_{9} } \right\}$, respectively, **e** initial tree with random mutation assignment for each ${\varvec{w}}_{d}$ to nodes of their corresponding paths, **f** result of sampling level for path ${\varvec{c}}_{1}$, **g** result of sampling level for path ${\varvec{c}}_{2}$, **h** result of sampling level for last two paths ${\varvec{c}}_{3}$ and ${\varvec{c}}_{4}$, **i** final tree after successive iterations of sampling path and sampling level

In order to make the sampling path clear, an example with defined inputs as single-cell mutation matrix $M$ and bulk data matrix $B$ is illustrated in Fig. 7. In this figure ${\varvec{w}}_{1} = \left\{ {M_{1} ,M_{2} ,M_{3} ,M_{4} ,M_{5} } \right\}$ is defined as a set of SNVs with the value of one in the first cell ($d = 1$) and ${\varvec{w}}_{2}$, ${\varvec{w}}_{3}$ and ${\varvec{w}}_{4}$ are defined for other cells similarly. In Fig. 7b it is shown that the first path of the tree corresponding to ${\varvec{w}}_{1}$ is generated with four levels and node labels are $\left\{ {\vartheta_{0} ,\vartheta_{1} ,\vartheta_{2} ,\vartheta_{3} } \right\}$. In Fig. 7c, the generated path for ${\varvec{w}}_{2}$ is shown. For generating this path, ${\varvec{w}}_{2}$ is first assigned to the node $\vartheta_{1}$ with the probability of $\frac{1}{\gamma + 1}$ and then, instead of assigning it to node $\vartheta_{2}$, a new node $\vartheta_{4}$ is generated with the probability of $\frac{\gamma }{{\gamma + 1{ }}}$. Generating new nodes continues up to level $k = 4$. The resulted tree after generating paths for ${\varvec{w}}_{3}$ and ${\varvec{w}}_{4}$ is shown in Fig. 7d. When the initial tree is generated, mutations of each ${\varvec{w}}_{d}$ are assigned to the nodes of their corresponding paths randomly as shown in Fig. 7e.

### Sampling level

Conifer clusters mutations for each path based on their VAFs similarity in different bulk sequencing samples. The possibility of having two distinct clones with the same mean value of VAFs makes their mutations clustering ambiguous. Considering the co-occurrence patterns of these mutations in single-cell sequencing data with their VAFs similarity is helpful to tackle this ambiguity. Therefore, Conifer performs sampling level with distance-dependent CRP instead of ordinary CRP of study Blei et al. [35] to consider the co-occurrence frequency of mutations as the distance between them. In other words, patterns of joining mutations resulted from distance-dependent CRP is the prior of Bayesian model which is based on single-cell data, and likelihoods of these patterns are computed by connectivity strength between clusters based on the VAFs of mutations in different bulk samples. Moreover, the posterior samples of SNVs level allocations for Gibbs sampler are summarized by the Maximum Posterior Expected Adjusted Rand (MPEAR) method [37]. Also, nodes are ordered according to the weighted average of their mean VAF value and mean value of the number of SNVs occurrence in a level (ISA assumption), so that nodes with the higher average mean value are placed in lower levels.

In the example of Fig. 7, the result of sampling level for path ${\varvec{c}}_{1}$ is shown in Fig. 7f in which two clusters with mutations $\left\{ {M_{1} ,M_{2} ,M_{3} } \right\}$ and $\left\{ {M_{4} ,M_{5} } \right\}$ are generated and node $\vartheta_{3}$ with no mutation is removed. Then, clustering is performed for mutations $\left\{ {M_{1} ,M_{2} ,M_{3} ,M_{9} ,M_{10} } \right\}$ in nodes $\vartheta_{1}$,${ }\vartheta_{4}$ and $\vartheta_{5}$ in the path ${\varvec{c}}_{2}$ and the node $\vartheta_{5}$ is removed and the resulted tree is shown in Fig. 7g. Result of sampling level for last two paths ${\varvec{c}}_{3}$ and ${\varvec{c}}_{4}$ is shown in Fig. 7h in which nodes $\vartheta_{7}$ and $\vartheta_{9}$ are removed as there is no mutation assigned to them. As it is shown in Fig. 7h, at the end of the sampling level, two nodes $\vartheta_{6}$ and $\vartheta_{1}$ present same genotype and will be merged together in the sampling path of the next iteration. Sampling path and sampling level steps are performed iteratively resulting in the final tree which is shown in Fig. 7i.

To explain how the co-occurrence frequency of mutations is calculated to be used in distance-dependent CRP, a distinct path ${\varvec{c}}_{d}$ is considered. The co-occurrence frequency of all pairs of SNVs in the path ${\varvec{c}}_{d}$ is defined by a $n_{{{\varvec{c}}_{d} }} \times n_{{{\varvec{c}}_{d} }}$ matrix ${\varvec{t}}_{{{\varvec{c}}_{d} }}$ which $n_{{{\varvec{c}}_{d} }} { }$ is the number of SNVs in the path ${\varvec{c}}_{d}$. Each element of ${\varvec{t}}_{{{\varvec{c}}_{d} }}$ is calculated by the number of cells that both SNVs have occurred in divided by the total number of cells in single-cell matrix ${\varvec{M}}$.

To cluster SNVs based on the similarity of VAFs, a $n_{{{\varvec{c}}_{d} }} \times n_{{{\varvec{c}}_{d} }}$ connectivity matrix ${\varvec{V}}_{{{\varvec{c}}_{d} }}$ is calculated for each distinct path ${\varvec{c}}_{d}$ in the tree. Each element of ${\varvec{V}}_{{{\varvec{c}}_{d} }}$ is the Euclidean distance of VAFs for the corresponding SNV pair in the path.

**Fig. 8:**
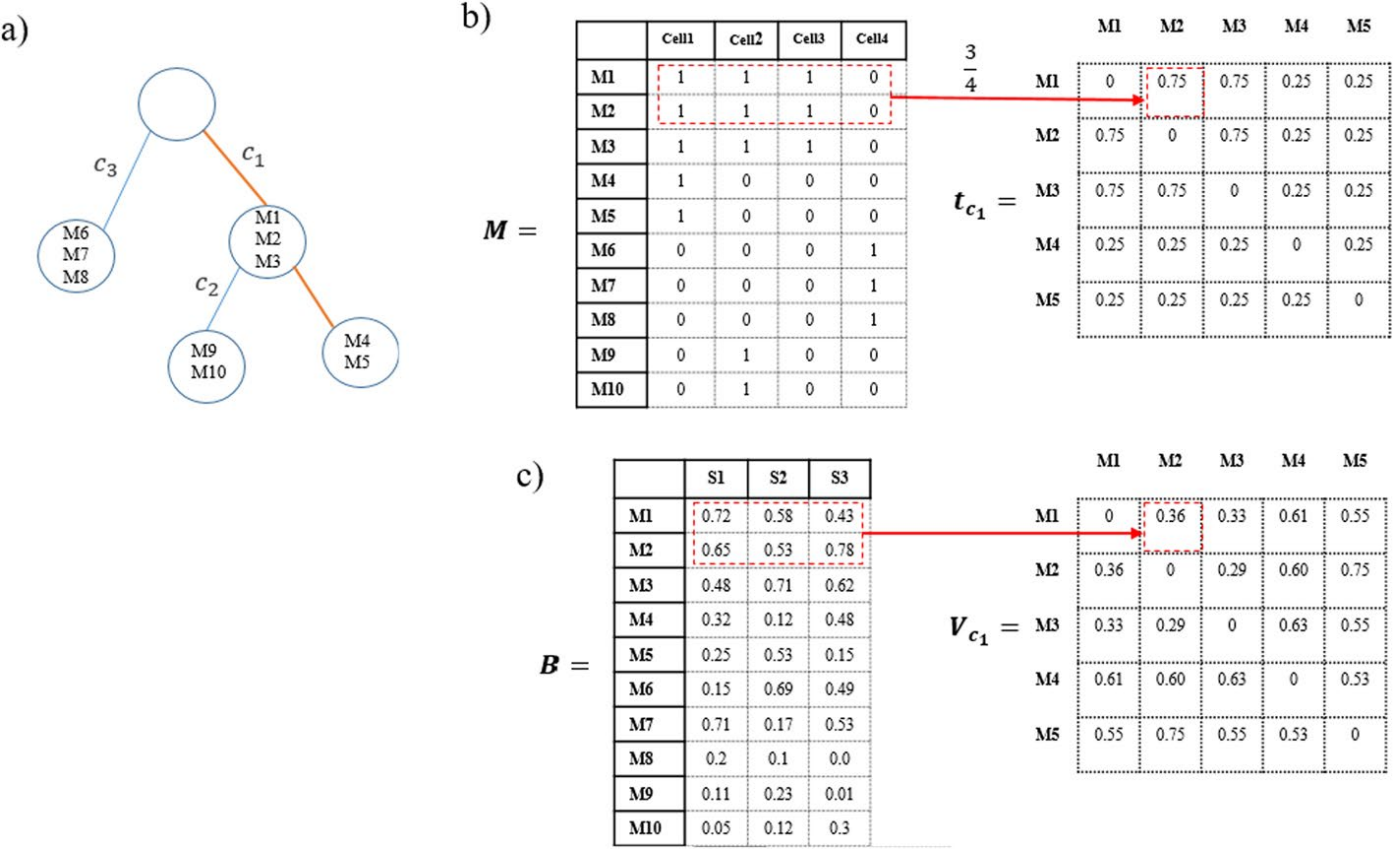
**a** A schematic example showing a clonal tree,** b** calculation of co-occurrence frequency of mutations ${\varvec{t}}_{{{\varvec{c}}_{1} }}$ for path ${\varvec{c}}_{1}$ in the clonal tree, **c** calculation of connectivity matrix ${\varvec{V}}_{{{\varvec{c}}_{1} }}$ for path ${\varvec{c}}_{1}$ in the clonal tree

As an example, the calculation of the co-occurrence frequency matrix ${\varvec{t}}_{{{\varvec{c}}_{1} }}$ and the connectivity matrix ${\varvec{V}}_{{{\varvec{c}}_{d} }}$ for the sample inputs of Fig. 7 is shown in Fig. 8.

### Formulation

Using the notation in the study of Baldassano et al. [38] for the connectivity clustering model, Conifer’s generative model formulation is described as follows:

3 $${\varvec{c}}_{d} { }\sim { }nCRP\left( {\gamma ,{\varvec{w}}_{d} } \right)\quad {\text{Nested}}\;{\text{CRP}}\;\left( {{\text{Sampling}}\;{\text{path}}} \right)$$

4 $${\varvec{z}}_{{{\varvec{c}}_{d} { }}} \sim { }ddCRP\left( {\eta ,{\varvec{f}},{\varvec{t}}_{{{\varvec{c}}_{d} }} ,{\varvec{V}}_{{{\varvec{c}}_{d} }} { }} \right)\quad {\text{Distance - dependent}}\;{\text{CRP}}\;\left( {{\text{Sampling}}\;{\text{level}}} \right)$$

5 $${\varvec{V}}_{{{\varvec{c}}_{d} ,ij}} \sim Normal\left( {{\varvec{A}}_{{l\left( {{\varvec{z}}_{{{\varvec{c}}_{d} { }}} } \right)_{i} { },{ }l\left( {{\varvec{z}}_{{{\varvec{c}}_{d} { }}} } \right)_{j} }} ,{\varvec{\sigma}}_{{l\left( {{\varvec{z}}_{{{\varvec{c}}_{d} { }}} } \right)_{i} { },{ }l\left( {{\varvec{z}}_{{{\varvec{c}}_{d} { }}} } \right)_{j} }}^{2} } \right)$$

6 $${\varvec{A}}_{{l\left( {{\varvec{z}}_{{{\varvec{c}}_{d} { }}} } \right)_{i} { },{ }l\left( {{\varvec{z}}_{{{\varvec{c}}_{d} { }}} } \right)_{j} }} ,{\varvec{\sigma}}_{{l\left( {{\varvec{z}}_{{{\varvec{c}}_{d} { }}} } \right)_{i} { },{ }l\left( {{\varvec{z}}_{{{\varvec{c}}_{d} { }}} } \right)_{j} }}^{2} { } \sim { }Normal - Inverse - \chi^{2} { }\left( {{\upmu }_{0} { },\kappa_{0} ,\sigma_{0}^{2} ,\nu_{0} } \right)$$

7 $$f_{ij} = \frac{{\exp \left( { - t_{{{\varvec{c}}_{d} ,ij}} { } + a} \right)}}{{\left( {1 + exp{ }\left( { - t_{{{\varvec{c}}_{d} ,ij}} { } + a} \right){ }} \right)}}$$

In this model ${\varvec{w}}_{d}$ is defined as a set of SNVs with the value of one in cell $d\left( {d = 1{ }to{ }m} \right)$ and ${\varvec{c}}_{d}$ is its corresponding path generated by nested CRP with a parameter $\gamma$ following Gamma distribution. ${\varvec{z}}_{{{\varvec{c}}_{d} { }}}$ is a vector with the size of the number of mutations in the path ${\varvec{c}}_{d}$ which is generated by distance-dependent CRP. It defines the mutation links for all mutations in the path ${\varvec{c}}_{d}$. Also, $l\left( {{\varvec{z}}_{{{\varvec{c}}_{d} { }}} } \right)$ is the level assignment derived from the ${\varvec{z}}_{{{\varvec{c}}_{d} { }}}$, for each mutation in the path ${\varvec{c}}_{d}$.

Moreover, $\eta$ is the model parameter following Gamma distribution and controls the self-loop in the connectivity graph. The decay function is represented by ${\varvec{f}}$ and the hyper-parameter $a$. The variable ${\varvec{t}}_{{{\varvec{c}}_{d} }}$ denotes the co-occurrence frequency matrix of all SNV pairs in the path ${\varvec{c}}_{d}$. In addition, ${\varvec{A}}$ denotes the connectivity strength of two clusters $l\left( {{\varvec{z}}_{{{\varvec{c}}_{d} { }}} } \right)_{i}$ and $l\left( {{\varvec{z}}_{{{\varvec{c}}_{d} { }}} } \right)_{j}$ and ${\varvec{\sigma}}^{2} { }$ is their connectivity variance. ${\varvec{A}}$ and ${\varvec{\sigma}}^{2}$ follow the $Normal - Inverse - \chi^{2} { }$ distribution function with scalar prior mean and precision of $\left( {{\upmu }_{0} ,\kappa_{0} } \right)$ and $\left( {\sigma_{0}^{2} ,\nu_{0} } \right)$, respectively.

Conifer probabilistic graphical model (Additional file 1: Fig. S7) and the table of notation reference (Additional file 1: Table S1) are provided in Additional file 1.

### Inference

Clonal tree of the tumor heterogeneity is found by posterior distribution inference on the path and level assignment of mutations from single-cell and bulk sequencing data, which is shown by $p({\varvec{c}}_{d} ,{\varvec{z}}_{{{\varvec{c}}_{d} { }}} |\gamma ,\eta ,{\varvec{f}},{\varvec{t}}_{{{\varvec{c}}_{d} }} ,{\varvec{V}}_{{{\varvec{c}}_{d} }} ,{\varvec{w}}_{d} )$. This posterior is approximated with Collapsed Gibbs sampling by iteratively performing sampling paths and sampling level assignments.

1. Sampling path:
  8 $$p\left( {{\varvec{c}}_{d} {|}{\varvec{c}}_{ - d} ,{\varvec{w}},{\varvec{z}},\gamma ,\eta } \right) \propto p\left( {{\varvec{c}}_{d} {|}{\varvec{c}}_{ - d} ,\gamma } \right)p\left( {{\varvec{w}}_{d} {|}{\varvec{c}},{\varvec{w}}_{ - d} ,{\varvec{z}},\eta } \right)$$
  In Eq. (8) which represents a Bayesian model, ${\varvec{c}}_{ - d}$ all paths existing in the tree after removing the mutations in the path corresponding to the cell $d$. The term $p\left( {{\varvec{w}}_{d} {|}{\varvec{c}},{\varvec{w}}_{ - d} ,{\varvec{z}},\eta } \right)$ represents the probability that ${\varvec{w}}_{d}$ has created a specific path, and $p\left( {{\varvec{c}}_{d} {|}{\varvec{c}}_{ - d} ,\gamma } \right){ }$ is the probability of prior which is based on the nested CRP and can be calculated by Eq. (1). The computational details are provided in Additional file 1.
2. Sampling level assignments:
  Conifer performs sampling on the mutation links ${{\varvec{z}}}_{d}$ as follows:
  9 $$\begin{aligned} & p\left( {\left( {z_{{{\varvec{c}}_{d} { }}} } \right)_{i}^{{\left( {new} \right)}} {|}\left( {{\varvec{z}}_{{{\varvec{c}}_{d} { }}} } \right)_{ - i} ,{\varvec{c}}_{d} ,{\varvec{V}}_{{{\varvec{c}}_{d} }} ,\eta ,{\varvec{f}},{\mathbf{t}}_{{{\mathbf{c}}_{{\text{d}}} }} } \right) \propto \\ & \quad \quad \quad p\left( {\left( {z_{{{\varvec{c}}_{d} { }}} } \right)_{i}^{{\left( {new} \right)}} {|}\eta ,{\varvec{f}},{\varvec{t}}_{{{\varvec{c}}_{d} }} } \right)p{(}{\varvec{V}}_{{{\varvec{c}}_{d} }} {|} l\left( {\left( {{\varvec{z}}_{{{\varvec{c}}_{d} { }}} } \right)_{ - i} \cup \left( {z_{{{\varvec{c}}_{d} }} } \right)_{i}^{{\left( {new} \right)}} } \right),{\varvec{c}}_{d} ) \\ \end{aligned}$$

In the Bayesian model of Eq. (9), $\left( {z_{{{\varvec{c}}_{d} }} } \right)_{i}$ denotes a link to mutation $i$ and $\left( {{\varvec{z}}_{{{\varvec{c}}_{d} }} } \right)_{ - i}$ is the vector of mutation links from which $\left( {z_{{{\varvec{c}}_{d} }} } \right)_{i}$ is removed. For considering different choices for sampling, the notation $\left( {z_{{{\varvec{c}}_{d} }} } \right)_{i}^{{\left( {new} \right)}}$ is used to denote a new link to mutation $i$ after removing $\left( {z_{{{\varvec{c}}_{d} }} } \right)_{i}$. In Eq. (9), the term $p\left( {\left( {z_{{{\varvec{c}}_{d} }} } \right)_{i}^{{\left( {new} \right)}} {|}\eta ,{\varvec{f}},{\varvec{t}}_{{{\varvec{c}}_{d} }} } \right)$ is the probability of prior which is based on the distance-dependent CRP and can be calculated by Eq. (2). The term $p{(}{\varvec{V}}_{{{\varvec{c}}_{d} }} {|} l\left( {\left( {{\varvec{z}}_{{{\varvec{c}}_{d} }} } \right)_{ - i} \cup \left( {z_{{{\varvec{c}}_{d} }} } \right)_{i}^{{\left( {new} \right)}} } \right),{\varvec{c}}_{d} )$ is the likelihood of ${\varvec{V}}_{{{\varvec{c}}_{d} }}$ according to the clusters given by $l\left( {\left( {{\varvec{z}}_{{{\varvec{c}}_{d} }} } \right)_{ - i} \cup \left( {z_{{{\varvec{c}}_{d} }} } \right)_{i}^{{\left( {new} \right)}} } \right)$ in the path ${\varvec{c}}_{d}$. The computational details are in Additional file 1.

## Supplementary Information

**Additional file 1**. Gibbs Sampling algorithm formulations.

## Data Availability

The sequencing datasets analyzed during the current study are available from the Sequence Read Archive with the accession number SRP074289 (for patient CRC2) and SRA053195 (for TNBC patients). All the source codes are available in the following link: https://github.com/LeilaBagha/Conifer.

## Abbreviations

DNA: Deoxyribonucleic acid
CRP: Chinese Restaurant Process
CNV: Copy number variation
MCMC: Markov chain Monte Carlo
MPEAR: Maximum Posterior Expected Adjusted Rand
VAF: Variant allele frequency
SNV: Single nucleotide variation
ISA: Infinite site assumption
