# Supplementary material for: Conifer: clonal tree inference for tumor heterogeneity with single-cell and bulk sequencing data
Source: BMC Bioinformatics. 2021 Aug 30;22:416. doi: 10.1186/s12859-021-04338-7 (PMC8404257; doi:10.1186/s12859-021-04338-7)
Supplement: Supplementary file 1 — Additional file 1. Gibbs Sampling algorithm formulations. [file 12859_2021_4338_MOESM1_ESM.docx]

**Supplementary material for**

**Conifer:** **Clonal Tree Inference for Tumor Heterogeneity with Single-Cell and Bulk Sequencing Data**

**Leila Baghaarabani**

Supplemental Figures


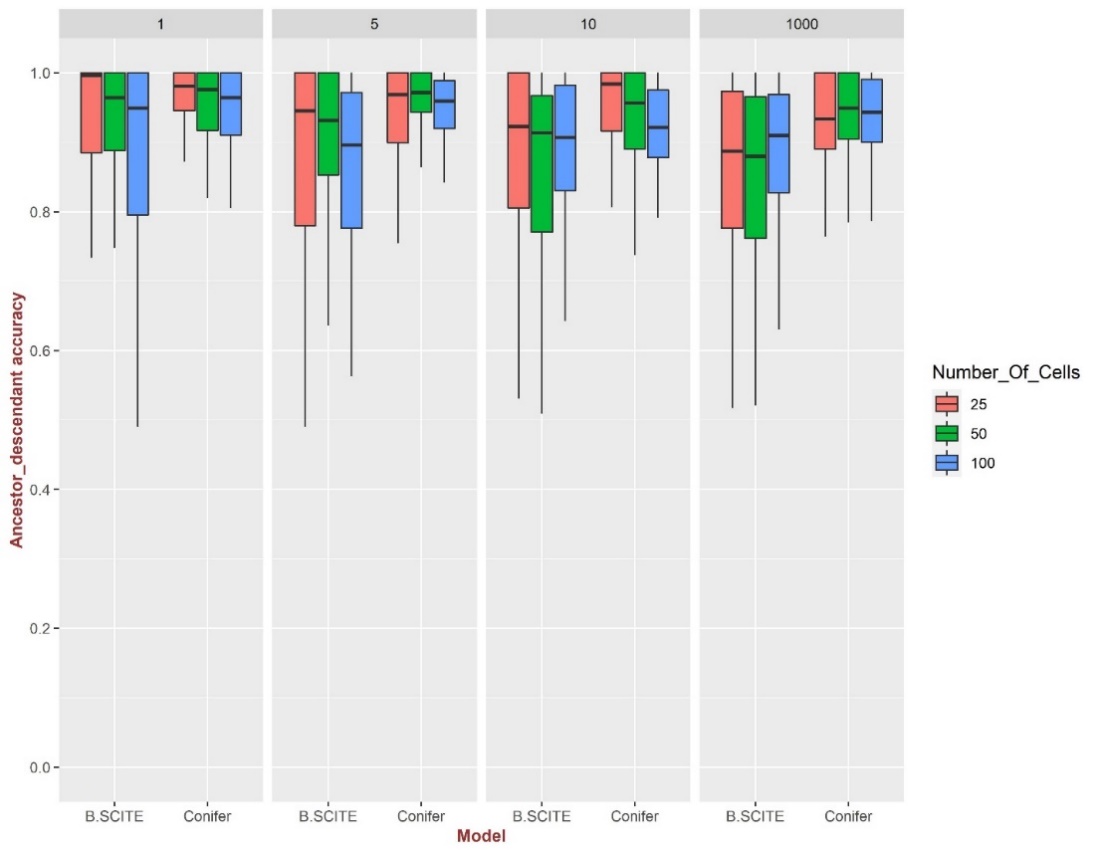


**Fig. S1** Comparison between mutation clustering accuracy of B-SCITE and Conifer on the simulated dataset with 30% of CNV, for 100 clonal trees simulated with 10 clones and 50 mutations. For $\lambda$ = 1, 5, 10 and 1000. For single-cell data, 50 genotypes are extracted for each clonal tree. There is one bulk sequencing sample with the coverage of 10^6^. The following errors are added to the single-cell set: the false-positive rate of ${10}^{-5}$, the false-negative rate of 0.2, missing rate of 0.05, and doublet rate of 0.1


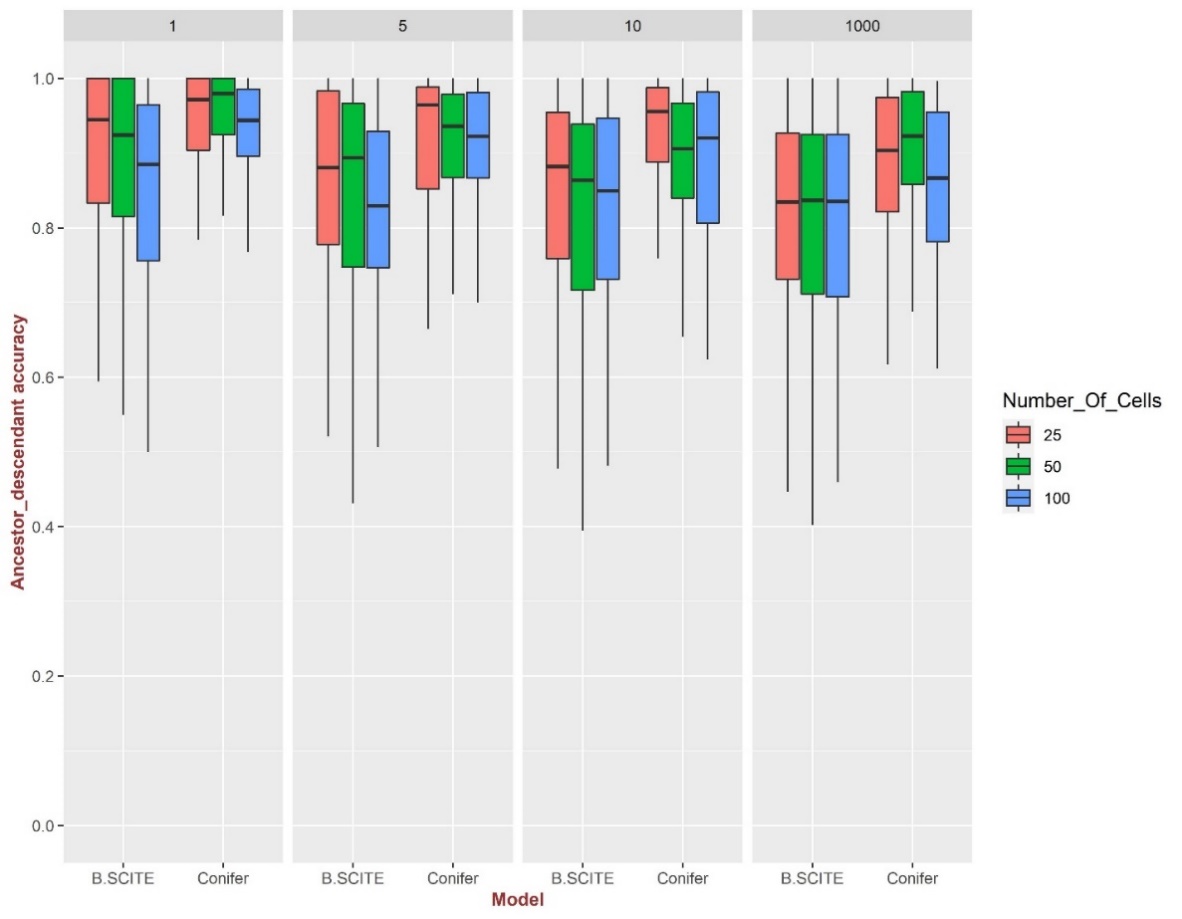


**Fig. S2** Comparison between mutation clustering accuracy of B-SCITE and Conifer on the simulated dataset with 50% of CNV, for 100 clonal trees simulated with 10 clones and 50 mutations. For λ = 1, 5, 10 and 1000. For single-cell data, 50 genotypes are extracted for each clonal tree. There is one bulk sequencing sample with the coverage of 106. The following errors are added to the single-cell set: the false-positive rate of ${10}^{-5}$, the false-negative rate of 0.2, missing rate of 0.05, and doublet rate of 0.1


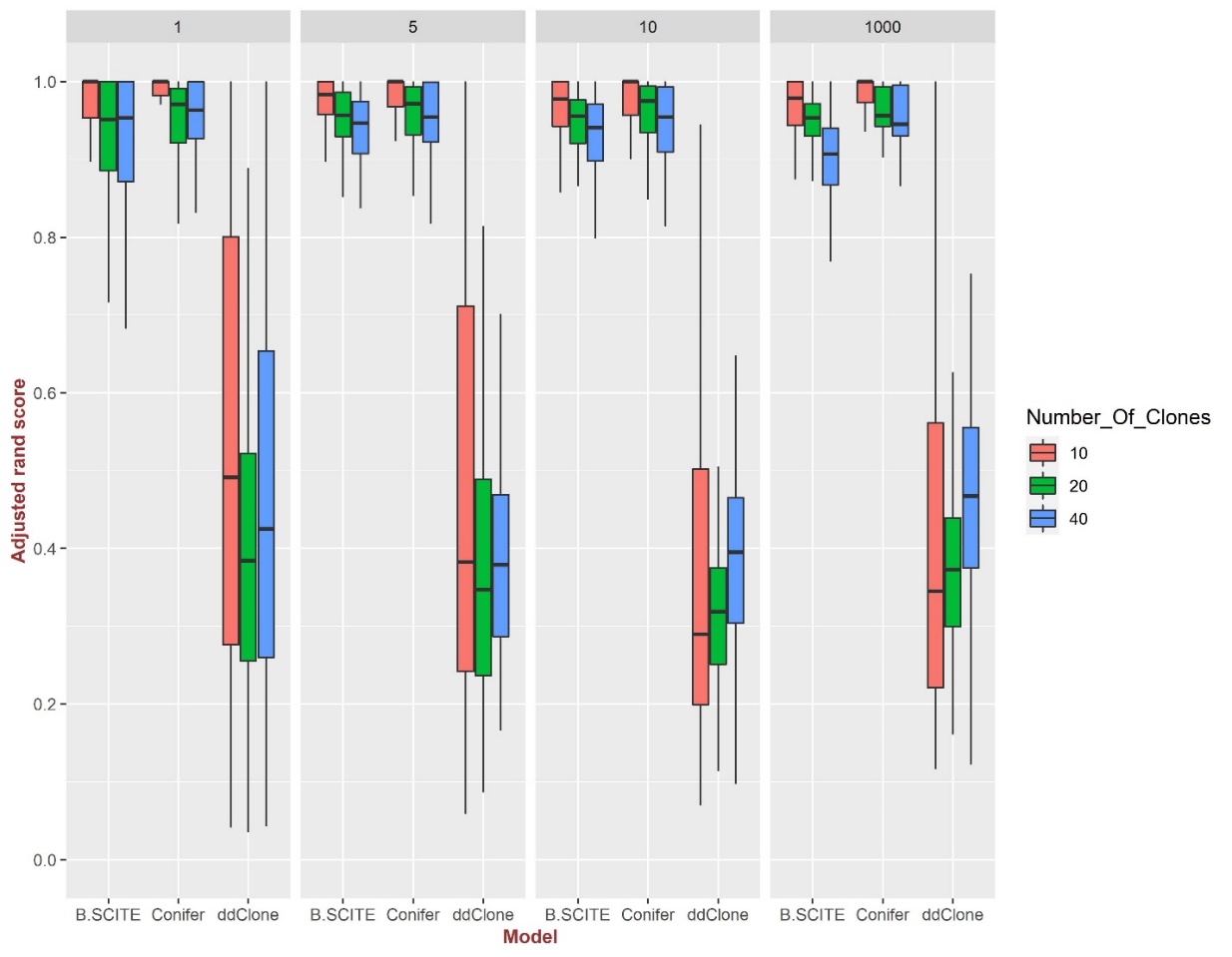


**Fig. S3** Comparison of mutation clustering accuracy in ddClone, B-SCITE, and Conifer for 100 clonal trees simulated with 10, 20, and 40 clones and 100 mutations. For $\lambda$ = 1, 5, 10 and 1000. For single-cell data, 50 genotypes are extracted for each clonal tree. There is one bulk sequencing sample with the coverage of 10,000. The following errors are added to the single-cell set: the false-positive rate of ${10}^{-5}$, the false-negative rate of 0.2, missing rate of 0.05, and doublet rate of 0.1.


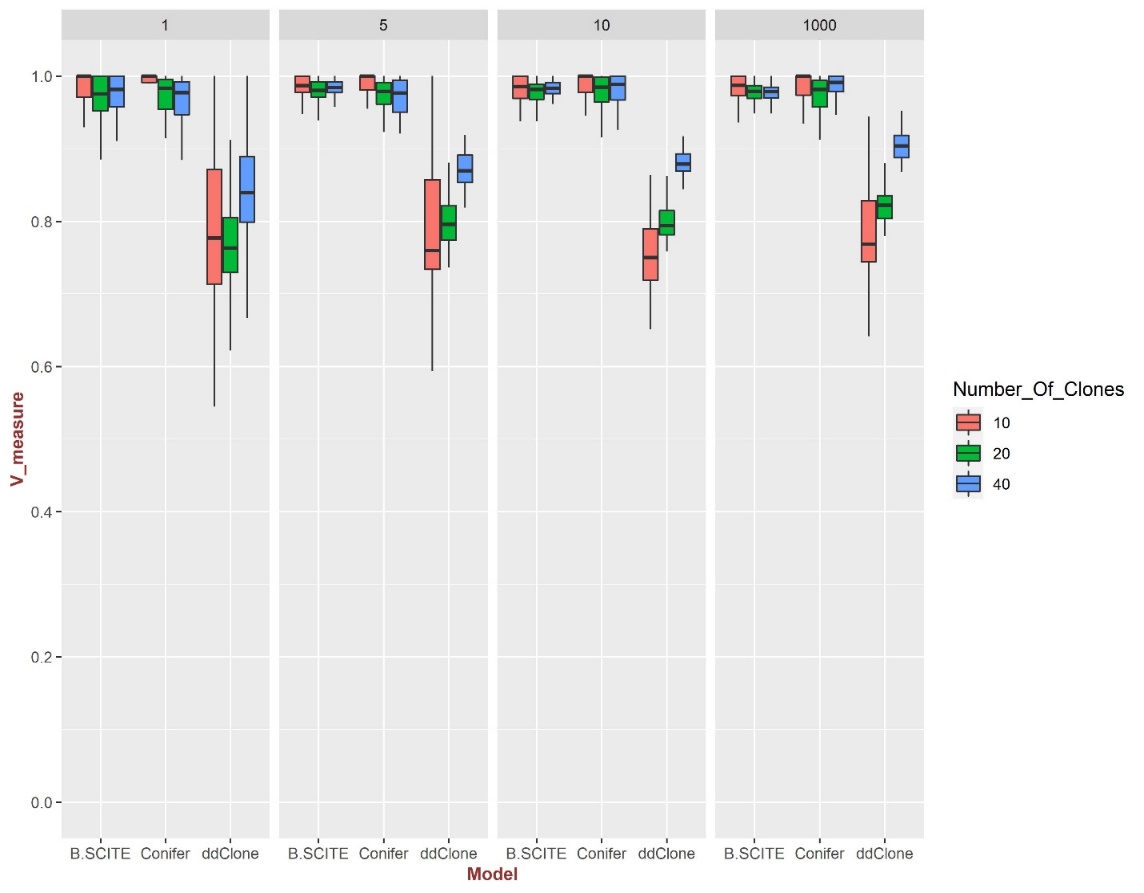


**Fig. S4** Comparison of mutation clustering accuracy in ddClone, B-SCITE, and Conifer methods for 100 clonal trees simulated with 10, 20, and 40 clones and 100 mutations. For $\lambda$ = 1, 5, 10 and 1000. For single-cell data, 50 genotypes are extracted for each clonal tree. There is one bulk sequencing sample with the coverage of 10,000. The following errors are added to the single-cell set: the false-positive rate of ${10}^{-5}$, the false-negative rate of 0.2, missing rate of 0.05, and doublet rate of 0.1.


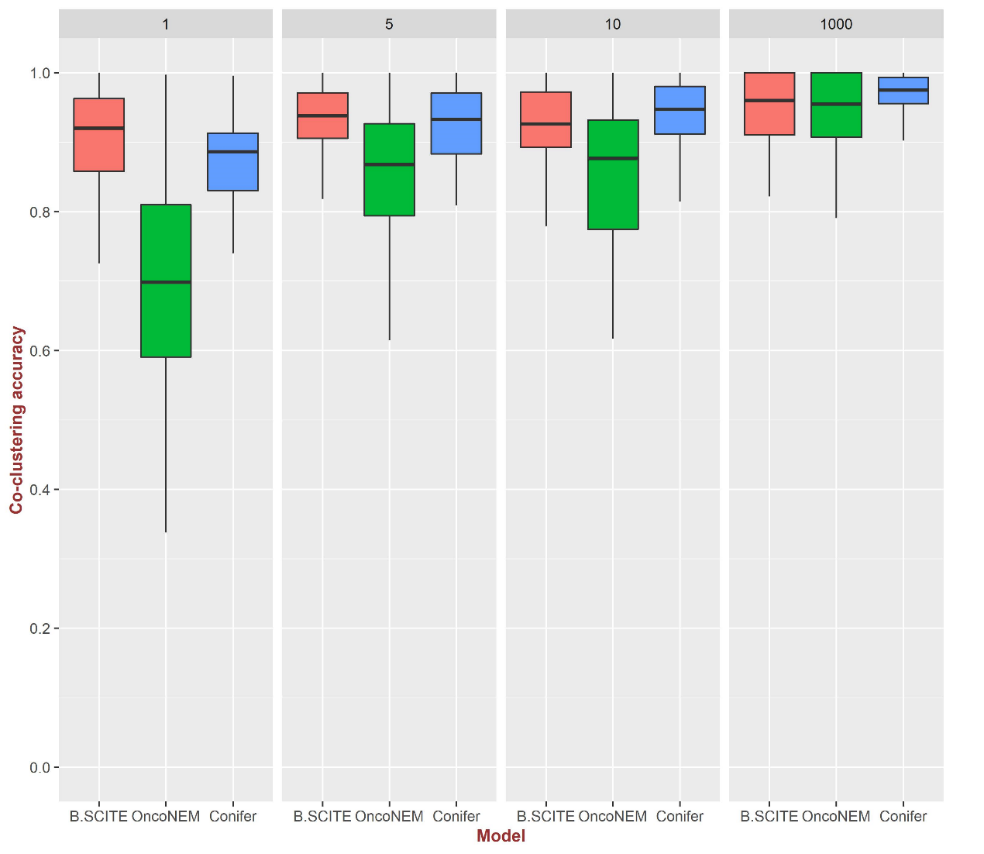


**Fig. S5** Comparison of tree inference between B-SCITE, OncoNEM, and Conifer for the false-positive rate of 0.01 and doublet rate of 0.1. For single-cell data, 50 genotypes are extracted for each clonal tree. There is one bulk sequencing sample with the coverage of 10,000. 100 clonal trees simulated with 10 clones and 50 mutations. For $\lambda$ = 1,5,10 and 1000. The following errors are added to the single-cell the false-negative rate of 0.2, missing rate of 0.05.


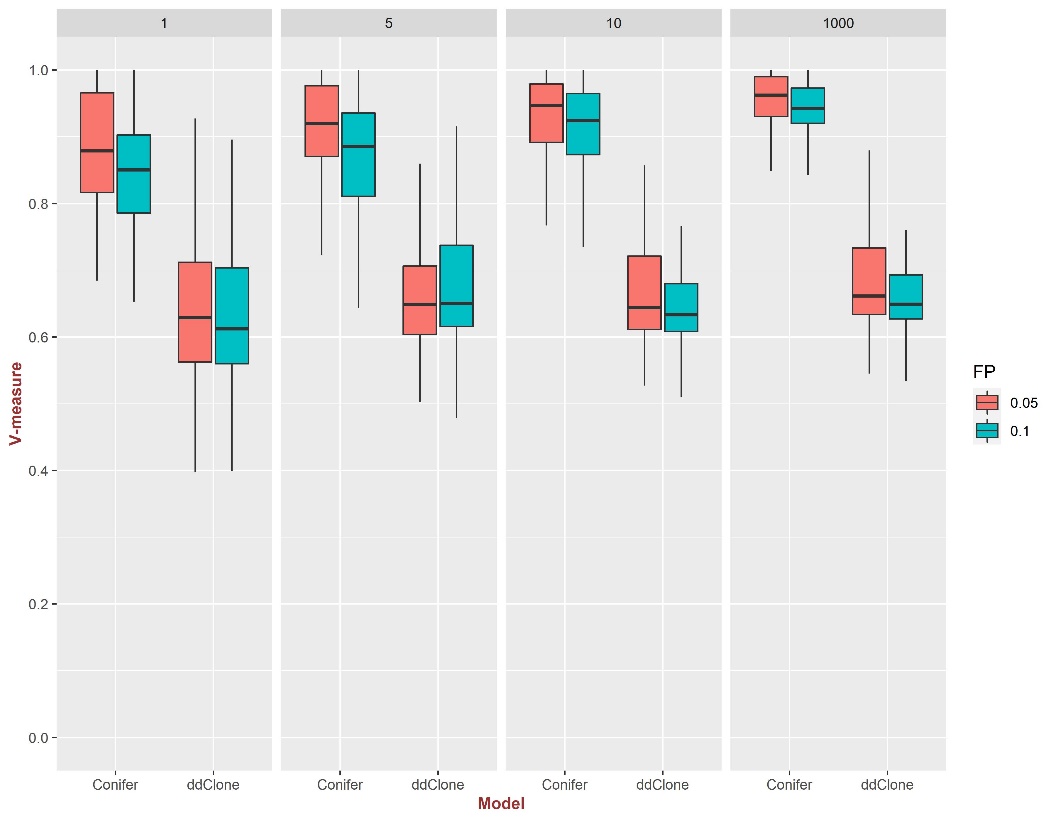


**Fig. S6** Comparison of clustering accuracy between Conifer and ddClone methods for false-positive rates of 5% and 10% and doublet rate of 0.05. For single-cell data, 500 genotypes are extracted for each clonal tree. There is one bulk sequencing sample with the coverage of 10,000. 50 clonal trees simulated with 10 clones and 50 mutations. For $\lambda$ = 1,5,10 and 1000. The following errors are added to the single-cell the false-negative rate of 0.2, missing rate of 0.05.


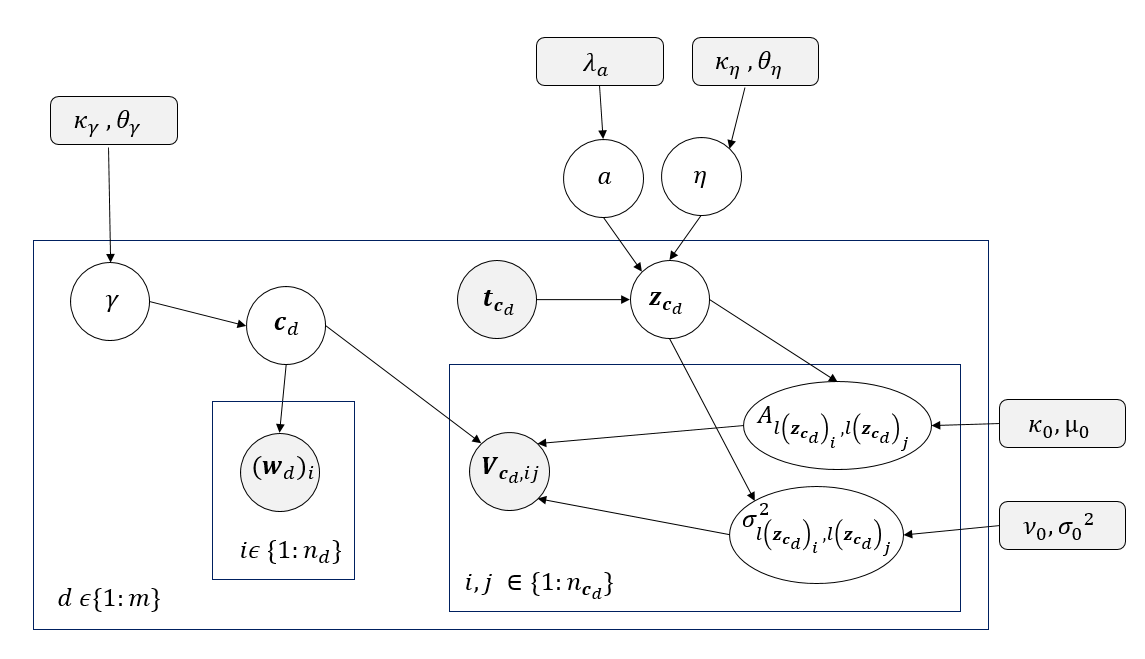


**Fig. S7** Probabilistic graphical model of Conifer. $\boldsymbol{w}_{d}$ is a set of SNVs with the size of $n_{d}$ with value of one in cell $d(d=1 to m)$ and $\boldsymbol{c}_{d}$ is its corresponding path generated by nested CRP with a parameter $\gamma$. $\boldsymbol{z}_{\boldsymbol{c}_{d}}$ is a vector with length equal to the number of mutations in the path $\boldsymbol{c}_{d}$ which is generated by distance-dependent CRP. $l(\boldsymbol{z}_{\boldsymbol{c}_{d}})$ is the level assignment derived from the $\boldsymbol{z}_{\boldsymbol{c}_{d}}$, for each mutation in the path $\boldsymbol{c}_{d}$. $\boldsymbol{A}$ denotes the connectivity strength of two clusters and $\boldsymbol{\sigma}^{2}$is their connectivity variance. $\boldsymbol{V}_{\boldsymbol{c}_{d},ij}$ is the connectivity matrix for the SNVs in the path $\boldsymbol{c}_{d}$ , and $n_{\boldsymbol{c}_{d}}$ is the number of SNVs in the path.

**Gibbs Sampling algorithm formulations**

1. Sampling path:

| $p\left( \boldsymbol{c}_{d} \vert\boldsymbol{c}_{-d},\boldsymbol{w},\boldsymbol{z},\gamma,\eta\right)\propto p\left( \boldsymbol{c}_{d} \vert\boldsymbol{c}_{-d},\gamma\right)p\left( \boldsymbol{w}_{d} \vert\boldsymbol{c},\boldsymbol{w}_{-d},\boldsymbol{z},\eta\right)$ | (S1) |
| --- | --- |
| $p\left( \boldsymbol{w}_{d} \vert\boldsymbol{c},\boldsymbol{w}_{-d},\boldsymbol{z},\eta\right)= \prod_{k=1}^{max{(\boldsymbol{z}}_{\boldsymbol{c}_{d}})} \left( \frac{\Gamma(\phi_{{{(\boldsymbol{z}}_{\boldsymbol{c}_{d}})}_{k},-d}\left( . \right)+N\eta)}{\prod_{w} \Gamma(\phi_{{{(\boldsymbol{z}}_{\boldsymbol{c}_{d}})}_{k},-d}\left( w \right)+\eta)} \frac{\prod_{w} \Gamma(\phi_{{{(\boldsymbol{z}}_{\boldsymbol{c}_{d}})}_{k},-d}\left( w \right)+\phi_{{{(\boldsymbol{z}}_{\boldsymbol{c}_{d}})}_{k},d}\left( w \right)+\eta)}{\Gamma(\phi_{{{(\boldsymbol{z}}_{\boldsymbol{c}_{d}})}_{k},-d}\left( . \right)+\phi_{{{(\boldsymbol{z}}_{\boldsymbol{c}_{d}})}_{k},d}\left( . \right)+N\eta)} \right)$ | (S2) |

Equation (S1) which is according to the study of Blei et al. [1] represents a Bayesian model and $\mathbf{c}_{-d}$ denotes all paths existing in the tree after removing the mutations in the path corresponding to the cell $d$. The term $p\left( \boldsymbol{w}_{d} | \boldsymbol{c},\boldsymbol{w}_{-d},\boldsymbol{z},\eta\right)$ represents the probability that $\boldsymbol{w}_{d}$ has created a specific path, and $p\left( \boldsymbol{c}_{d} | \boldsymbol{c}_{-d},\gamma\right)$ is the probability of prior which is based on the nested CRP. The standard Gamma function is shown by $\Gamma$ and $N$ is the total number of SNVs. $\phi_{{{(\mathbf{z}}_{c_{d}})}_{k},-d}\left( w \right)$ denotes the number of instances of the mutation $w$ which is assigned to the clone with index ${{(\mathbf{z}}_{\mathbf{c}_{d}})}_{k}$ and is not in the cell $d$. $\phi_{{{(\mathbf{z}}_{\mathbf{c}_{d}})}_{k},-d}\left( . \right)$ denotes the total number of mutations that are assigned to clone with index ${{(\mathbf{z}}_{\mathbf{c}_{d}})}_{k}$and are not in cell $d$ .

1. Sampling level

| $p\left( \left( z_{\boldsymbol{c}_{d}} \right)_{i}^{\left( new \right)} \vert\left( \boldsymbol{z}_{\boldsymbol{c}_{d}} \right)_{-i},\boldsymbol{c}_{d},\boldsymbol{V}_{\boldsymbol{c}_{d}},\eta,\boldsymbol{f},\boldsymbol{t}_{\boldsymbol{c}_{d}} \right) \propto p\left( \left( z_{\boldsymbol{c}_{d}} \right)_{i}^{\left( new \right)} \vert\eta,\boldsymbol{f},\boldsymbol{t}_{\boldsymbol{c}_{d}} \right)p\left( \boldsymbol{V}_{\boldsymbol{c}_{d}} \right\vert l\left( \left( \boldsymbol{z}_{\boldsymbol{c}_{d}} \right)_{-i}\cup\left( z_{\boldsymbol{c}_{d}} \right)_{i}^{\left( new \right)} \right),\boldsymbol{c}_{d})$ | (S3) |
| --- | --- |
| $p\left( \boldsymbol{V}_{\boldsymbol{c}_{d}} \right\vert l\left( \left( \boldsymbol{z}_{\boldsymbol{c}_{d}} \right)_{-i}\cup\left( z_{\boldsymbol{c}_{d}} \right)_{i}^{\left( new \right)} \right),\boldsymbol{c}_{d} )\propto\prod_{k_{1},k_{2}=1}^{\max\left( l\left( \boldsymbol{z}_{\boldsymbol{c}_{d}} \right) \right)} p\left( {{(\boldsymbol{V}}_{\boldsymbol{c}_{d}})}_{l_{k_{1}}^{\left( new \right)},l_{k_{2}}^{\left( new \right)}} \right)$ | (S4) |
| $\frac{p(\boldsymbol{V}_{\boldsymbol{c}_{d}}\vert\hat{l})}{p(\boldsymbol{V}_{\boldsymbol{c}_{d}}\vert l)}= \frac{\prod_{k=1}^{K} p({{(\boldsymbol{V}}_{\boldsymbol{c}_{d}})}_{\hat{l}_{k},\hat{l}_{K}})\prod_{k=1}^{K-1} p({{(\boldsymbol{V}}_{\boldsymbol{c}_{d}})}_{\hat{l}_{K},\hat{l}_{k}})}{\prod_{k=1}^{K^{'}} p({{(\boldsymbol{V}}_{\boldsymbol{c}_{d}})}_{l_{k},l_{K^{'}}})\prod_{k=1}^{K^{''}} p({{(\boldsymbol{V}}_{\boldsymbol{c}_{d}})}_{l_{k},l_{K^{''}}})\prod_{k=1}^{K-1} p({{(\boldsymbol{V}}_{\boldsymbol{c}_{d}})}_{l_{K^{'}},l_{k}})\prod_{k=1}^{K^{'}} p({{(\boldsymbol{V}}_{\boldsymbol{c}_{d}})}_{l_{K^{''}},l_{k}})}$ | (S5) |

In the Bayesian model of equation (S3), $\left( z_{\boldsymbol{c}_{d}} \right)_{i}$ denotes a link to mutation $i$ and $\left( \boldsymbol{z}_{\boldsymbol{c}_{d}} \right)_{-i}$ is the vector of mutation links from which $\left( z_{\boldsymbol{c}_{d}} \right)_{i}$ is removed. For considering different choices for sampling, the notation $\left( z_{\boldsymbol{c}_{d}} \right)_{i}^{\left( new \right)}$ is used to denote a new link to mutation $i$ after removing $\left( z_{\boldsymbol{c}_{d}} \right)_{i}$. The term $p\left( \left( z_{\boldsymbol{c}_{d}} \right)_{i}^{\left( new \right)} | \eta,\boldsymbol{f},\boldsymbol{t}_{\boldsymbol{c}_{d}} \right)$ is the probability of prior which is based on the distance-dependent CRP and the term $p\left( \boldsymbol{V}_{\boldsymbol{c}_{d}} \right| l\left( \left( \boldsymbol{z}_{\boldsymbol{c}_{d}} \right)_{-i}\cup\left( z_{\boldsymbol{c}_{d}} \right)_{i}^{\left( new \right)} \right),\boldsymbol{c}_{d})$ is the likelihood of $\boldsymbol{V}_{\boldsymbol{c}_{d}}$ according to the clusters given by $l\left( \left( \boldsymbol{z}_{\boldsymbol{c}_{d}} \right)_{-i}\cup\left( z_{\boldsymbol{c}_{d}} \right)_{i}^{\left( new \right)} \right)$ in the path $\boldsymbol{c}_{d}$. If the $\left( z_{\boldsymbol{c}_{d}} \right)_{i}$ is resampled to mutation $i$ (self-loop) or the mutation $j$ in such a way that clusters do not change then ${l(\left( \boldsymbol{z}_{\boldsymbol{c}_{d}} \right)}_{-i})= l$.

Otherwise, if resampling of $\left( z_{\boldsymbol{c}_{d}} \right)_{i}$ to the mutation $j$ results in merging clusters $K^{'}$ and $K^{''}$ in ${l(\left( \boldsymbol{z}_{\boldsymbol{c}_{d}} \right)}_{-i})$ and creation of cluster $K$ in $l\left( \left( \boldsymbol{z}_{\boldsymbol{c}_{d}} \right)_{-i}\cup\left( z_{\boldsymbol{c}_{d}} \right)_{i}^{\left( new \right)} \right)= \hat{l}$, then the probability of merging or splitting two clusters at each sampling step is computed by equation (S5) and by considering the numbering of clusters as $l_{i}\in\{1\ldots\left( K-1 \right),K^{'},K^{''}\}$ and $\hat{l}_{i}\in\{1\ldots\left( K-1 \right),K\}$. Each term $p({{(\boldsymbol{V}}_{\boldsymbol{c}_{d}})}_{l_{m},l_{n}})$ is the marginal likelihood of $Normal-Inverse-\chi^{2}$. More details are provided in the study of Baldassano et al. [2].

**Table S1** Notation reference for Conifer model

| **Variable** | **Description** | **Observed** |
| --- | --- | --- |
| $\boldsymbol{c}_{d}$ | The path generated by nested CRP for $\boldsymbol{w}_{d}$ | No |
| $\boldsymbol{w}_{d}$ | A set of SNVs with the value of one in cell $d$ | Yes |
| $\gamma$ | The parameter of nested CRP model with the distribution of $Gamma(\kappa_{\gamma}, \theta_{\gamma})$ | No |
| $\kappa_{\gamma}$ | Shape hyper-parameter over the parameter $\gamma$ | Yes |
| $\theta_{\gamma}$ | Rate hyper-parameter over the parameter $\gamma$ | Yes |
| $\boldsymbol{z}_{\boldsymbol{c}_{d}}$ | The vector of mutation links in the path $\boldsymbol{c}_{d}$ generated by distance-dependent CRP | No |
| $l(\boldsymbol{z}_{\boldsymbol{c}_{d}})$ | Level assignment derived from the $\boldsymbol{z}_{\boldsymbol{c}_{d}}$ | No |
| $\eta$ | The parameter of distance-dependent CRP model with the distribution of $Gamma(\kappa_{\eta}, \theta_{\eta})$ | No |
| $\kappa_{\eta}$ | Shape hyper-parameter over the parameter $\eta$ | Yes |
| $\theta_{\eta}$ | Rate hyper-parameter over the parameter $\eta$ | Yes |
| $\boldsymbol{f}$ | The decay function with hyper-parameter $a$ | No |
| $a$ | The parameter of $\boldsymbol{f}$ with the distribution of $Exponential(\lambda_{a})$ | No |
| $\lambda_{a}$ | Hyper-parameter for the parameter $a$ | Yes |
| $\boldsymbol{t}_{\boldsymbol{c}_{d}}$ | The co-occurrence frequency of mutations of path $\boldsymbol{c}_{d}$ | Yes |
| $\boldsymbol{V}_{\boldsymbol{c}_{d}}$ | The observed connectivity between the SNVs of the path $\boldsymbol{c}_{d}$ | Yes |
| $\boldsymbol{A}_{l_{1},l_{2}}$ | The connectivity strength of two clusters $l_{1}$ and $l_{2}$ | No |
| $\boldsymbol{\sigma}_{l_{1},l_{2}}^{2}$ | The connectivity variance of two clusters $l_{1}$ and $l_{2}$ | No |
| $\mu_{0}$ | The scalar prior mean for the connectivity strength | Yes |
| $\kappa_{0}$ | The precision for the connectivity strength | Yes |
| $\sigma_{0}$ | The scalar prior mean for the connectivity variance | Yes |
| $\nu_{0}$ | The precision for the connectivity variance | Yes |
| $n_{\mathbf{c}_{d}}$ | The number of mutations in the path $\mathbf{c}_{d}$ | No |
| $n_{d}$ | The number of SNVs with the value of one in cell $d$ | No |
| $n$ | The number of SNVs | Yes |
| $m$ | The number of cells | Yes |

**The idea of the Conifer model**

**Hierarchical topic model**: The objective of the hierarchical topic model in the study of Blei [3] is identifying subsets of words that co-occur within documents as topics and arranging them into a tree in such a way that more general topics are near to the root. Moreover, a document is a path in the tree, which is generated by the topics that are appeared on it.

**Conifer model description:** Following the hierarchical topic model idea, SNVs, clones, and clonal trees in the Conifer method correspond to words, topics, and topic hierarchy, respectively. In addition, a single-cell profile corresponds to a document that is generated by the clones on a path in the tree. In fact, each clone, which is a node in the tree, is a probability distribution on the SNVs, and a path is an infinite set of them. In Conifer, the Blei model [3] is extended in such a way that instead of ordinary CRP, distance-dependent CRP [4] is used in each node of the tree to define prior over its descendant.

Introducing a clonal tree in Conifer is based on identifying single-cell mutation profiles on the paths generated by the nested CRP. Conifer introduces a two-dimensional generative model that firstly, defines nodes as probability distributions over SNVs and secondly, defines a probability distribution on a set of nodes on each path in the tree.

Although the modification of Conifer on the Blei’s model [3] reduces the probability of repeated mutations on different nodes of tree significantly, however, it is not yet impossible. In order to satisfy the ISA assumption, the most appropriate clone for the repeated mutations can be inferred by a straightforward post-processing step in which the VAF of the repeated mutation in bulk sequencing sample is compared to mean VAF of mutations of all clones that the mutation belongs to and the clone with the least difference is selected as the clone for the repeated mutation.

**Details of generating simulated data**

For generating simulated data the ideas of ddClone [5] and B-SCITE [6] studies are used and briefly described here by the notation of B-SCITE [6]. Suppose that $n_{t}$ is the number of clonal trees which are generated randomly and each tree has $n_{c}$ clones (nodes) and $n$ is the number of mutations. The nodes of tree are labeled by $V=\{v_{1},\ldots,v_{n_{c}+1}\}$ as $v_{n_{c}+1}$ is the label of root node without mutation and other nodes are genetically different. Mutations are distributed between nodes so that no node is left without mutation except the root node. Cellular population frequencies for node $v_{i}$in bulk sample j are shown by $\Phi_{ij}$ with lower bound $0.02$ which is calculated as follows:

$$\Phi_{ij}= 0.02+[1-0.02\times\left( n_{c}+1 \right)]\times\frac{\omega_{ij}}{\sum_{l=1}^{n_{c}+1} \omega_{lj}}$$

The $\omega_{ij}$ is a random number between (0,1). Bulk sequencing read counts are drawn from the binomial distribution with parameter $t$ and success probability $\frac{y}{2}$ which $y$ is the cellular prevalence of mutation $M$ in the bulk sequencing sample. The cellular prevalence of mutation $M$ is calculated as the sum of frequencies of cellular populations possessing $M$ in that sample.

In sampling single-cell from the subclones genotype, it is important to consider sequencing errors like assortment bias. Assortment bias is a single-cell sequencing error that occurs when genotypes of sampled cells do not properly represent the genotypic distribution of the tumor cell population. To simulate assortment bias error in single-cell data, new genotype prevalence is obtained by sampling from a Dirichlet distribution with parameter *λ* on the average cell prevalence of all bulk sequencing data. Suppose $\psi_{i}$ is the average cellular prevalence of clone $v_{i}$ and the Dirichlet distribution is $\psi_{observed}=Dir(\lambda\psi)$. Large values of λ indicate less assortment bias and equivalently less difference between single-cell and bulk genotype prevalence. In this study, for measuring the sensitivity of the Conifer method to assortment bias, four sets of cells with different $\lambda$ ($\lambda$ =1, 5, 10, and 1000) are simulated.

Doublet is a type of error that occurs in single-cell sequencing data when one or more single cells are placed together in sequencing well, and consequently, their genotypes are mixed, and the signal of a genotype shows a greater mutant locus than each cell trapped in the well. They are considered as false-positive errors. For considering this type of error while simulating the single-cell data, it is unified with the next simulated cell with probability δ. For adding false positives and false negatives 0 and 1 are flipped to 1 and 0 with probability $\alpha$ and $\beta$, respectively. For more details of implementation refer to supplementary information of B-SCITE [6].

**Reference**

1. Blei DM, Griffiths TL, Jordan MI, Tenenbaum JB: **Hierarchical topic models and the nested Chinese restaurant process**. In: *NIPS: 2003*.

2. Baldassano C, Beck DM, Fei-Fei L: **Parcellating connectivity in spatial maps**. *PeerJ* 2015, **3**:e784.

3. Blei DM, Griffiths TL, Jordan MI: **The nested chinese restaurant process and bayesian nonparametric inference of topic hierarchies**. *Journal of the ACM (JACM)* 2010, **57**(2):1-30.

4. Blei DM, Frazier PI: **Distance dependent Chinese restaurant processes**. *Journal of Machine Learning Research* 2011, **12**(8).

5. Salehi S, Steif A, Roth A, Aparicio S, Bouchard-Côté A, Shah SP: **ddClone: joint statistical inference of clonal populations from single cell and bulk tumour sequencing data**. *Genome biology* 2017, **18**(1):1-18.

6. Malikic S, Jahn K, Kuipers J, Sahinalp SC, Beerenwinkel N: **Integrative inference of subclonal tumour evolution from single-cell and bulk sequencing data**. *Nature communications* 2019, **10**(1):1-12.
